# A new inertial navigation system for guiding implant placement. An in-vitro proof-of-concept study

**DOI:** 10.1371/journal.pone.0255481

**Published:** 2021-10-21

**Authors:** G. Esteve-Pardo, L. Esteve-Colomina, E. Fernández

**Affiliations:** 1 Bioengineering Institute, Miguel Hernández University of Elche, Group Aula Dental Avanzada, Alicante, Spain; 2 Bioengineering Institute, Miguel Hernández University of Elche and CIBER-BBN, Elche, Spain; International Medical University, MALAYSIA

## Abstract

The aim of this study was to assess the potential use of a new advanced inertial navigation system for guiding dental implant placement and to compare this approach with standard stereolithographic template guiding. A movement processing unit with a 9-axis absolute orientation sensor was adapted to a surgical handpiece and wired to a computer navigation interface. Sixty implants were placed by 10 operators in 20 jaw models. The 30 implants of the test group were placed in 10 models guided by the new inertial navigation prototype. The 30 implants of the control group were placed in another 10 models using a CAD-CAM template. Both groups were subdivided into experienced and non-experienced operators. Pre- and postoperative computer tomography images were obtained and matched to compare the planned and final implant positions. Four deviation parameters (global, angular, depth, and lateral deviation) were defined and calculated. The primary outcome was the angular deviation between the standard stereolithographic approach and the new inertial navigation system. Results showed no significant differences between both groups, suggesting that surgical navigation based on inertial measurement units (IMUs) could potentially be useful for guiding dental implant placement. However, more studies are still needed to translate this new approach into clinical practice.

## Introduction

It is generally believed that prosthetically driven implant placement is a prerequisite to achieve the precise positioning required for the final restoration [1]. The proper placement of implants is also necessary to prevent future technical and biological complications [2]. However, the accuracy of implant placement is inexorably subjected to the so-called “human factors” [3]. In this sense, virtual planning software and computer-assisted surgery have significantly improved the accurate transfer of implants from the pre-planned position to the final surgical position.

Computer-guided implant placement can be performed either statically via splints or by the dynamic navigation devices. Guiding by both computer-aided design and computer-aided manufacturing (CAD-CAM) surgical guides [4–8] or by dynamic navigation [9–13] was found to achieve a higher precision of the target implant position compared with conventional free-hand implant placement.

In the last few decades, several dynamic navigation systems based on different tracking technologies have been developed. Optical tracking systems are currently the most used in implant dentistry [12–14]. Navigation systems based on electromagnetic sensors are well established mostly in minimally invasive surgery [15], but their application to implant dentistry has the drawback of distortions from interference of neighbouring medical devices or ferromagnetic objects [16]. Hybrid devices combining both trackers in a magneto-optic hybrid system have been validated for maxillofacial surgery [17,18].

As in the case of template guiding, implant placement by surgical navigation has been shown to be more accurate, faster, and easier than conventional non-guided surgery [19]. Despite these benefits, the added cost, the complex ergonomics required, and the unavoidable learning curve [19] result in these technologies being dismissed in the clinical routine. According to market analysis (iData Research Inc.), in the US, computer-guide surgery will reach only 3.7% of the total projected 2021 implant market size [20].

The inertial measurement unit (IMU) is a combination of three sensors simultaneously detecting the velocity, orientation, and gravitational force of an object. The linear acceleration is measured by accelerometers, the rotational rate is measured by gyroscopes, and additionally the heading reference can be provided by a magnetometer that can be included in the same microchip. Micro Electro Mechanical Systems (MEMS) technology has successfully reduced the size of these sensors to a sub-millimetre scale, and their cost to the same extent.

IMUs have been widely used in navigation, to operate air vehicles, and in areas such as manufacturing and robotics. They still show a great potential for further developments and applicability due to their simplicity, miniature size, low cost, and easy of use [21]. The raw measurements of the three kinds of sensors are filtered and merged by the software to calculate attitude, angular rates, linear velocity, and relative position within a magnetic field. In short, the computer can follow the movement of the object in real time for each of the three axes known as pitch, roll, and yaw in a vehicle [22].

Until now, inertial sensing has been used in various hybrid surgical navigation systems. In these systems, IMUs functioned to compensate for electromagnetic sensor errors, or to support for partial occlusions in optical tracking devices. Preliminary clinical studies have been conducted in various surgical fields like endoscopic and laparoscopic surgery [23–25] and orthopedic surgery [26–29].

Due to their characteristics, IMUs can also be applicable to implant surgery. But, to our knowledge, its presumed possibilities have not yet been explored in this field, beyond an in vitro study [30].

The present study aims to validate the potential usefulness of IMU technology to assist in the placement of dental implants. The accuracy in transferring implants to the planned position with the help of an inertial device was evaluated and compared with a “gold-standard” control, the placement of implants using stereolithographic templates. Our results suggest that an inertial guide system could be as accurate as standard surgical templates.

## Materials and methods

Twenty identical jaw models were designed using Autodesk Inventor 2016 (Autodesk Inc.), manufactured with Fused Filament Fabrication (FFF) 3D printer (BCN3D Sigma R17) and polylactic acid (PLA) resin (BCN PLA 2,85 mm). Prints were made with 0,1 mm layer height and positioning resolution (X/Y/Z) of 12,5 μm/12,5 μm/1 μm.

Since maxillary models are more difficult to align we only used mandibular models. No attempt was made to emulate the anatomy in detail, since our main goal was to assess the precision of implant placement with respect to the planned positions. The models had an extra posterior space to place a digital inclinometer BDJK model SH-5339-90 (Shenzhen Jieshun Science and Technology Industry Co., Ltd). An inclinometer was used to assure the reproducibility during the scanning radiology and later during the drilling process at 0°. Three shallow holes were prepared in the models to facilitate the location of the planned osteotomies’ starting points. Furthermore, three triangular protrusions enabled a firm hold on the splint used for drilling and included two vertical pillars with concavities to position and calibrate the IMU adapter to the surgical handpiece.

**Fig 1:**
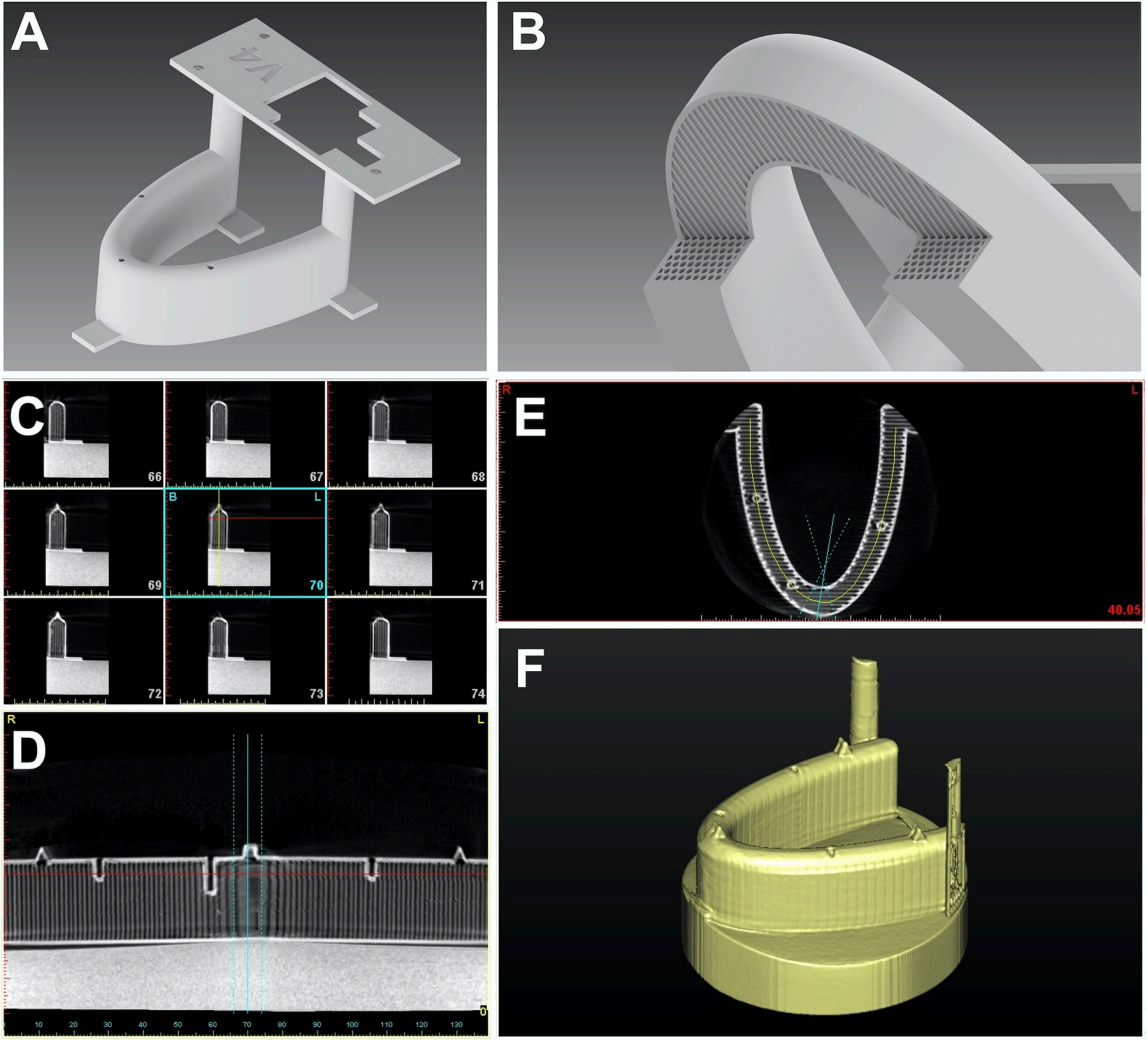
Different views of the jaw model. Initial digital design of the model (A) with the internal honeycomb design (B) to allow implant bed preparation and insertion. DICOM views (Sagittal (C), coronal (D), axial (E) and tridimensional rendering (F) from the jaw model prior to planning implant positions with the Simplant Pro v.17.01 software planner.

Computer scans of the models were obtained using cone beam computed tomography (CBCT) scans with the help of Planmeca ProMax 3D Plus (Planmeca Oy), in a 0° position and the following parameters: 90 kV, 10 mA, FOV 200 x 100 mm and 600 μm voxel size. Five hundred one slices were obtained per CBCT image and model. The digital imaging and communication on medicine (DICOM) files (Fig 1) were then exported to the software planner Simplant Pro v. 17.01 (Dentsply Sirona Inc.), where three 4.2 x 13 mm Dentsply Astra Tech EV implants were planned with different angulations. According to these planned parameters, a *S*implant Safe Guide was fabricated (Materialise NV). The drilling protocol of the Astra Tech Implant System EV was followed to place the implants into the models using the fully guiding template.

A high-performance orientation sensor, able to estimate 3D orientation in space, was used for real-time surgical navigation. The selected device was the Adafruit BNO 055 (Adafruit Industries), which contains a microprocessor and 3 specific microsensors: a triaxial 16-bit gyroscope, a triaxial 14-bit accelerometer, and a triaxial geomagnetic sensor, totalling 9 degrees of freedom (DoF). The microprocessor is a 32-bit a high-speed ARM Cortex-M0 microcontroller that collects raw data from the 3 sensors. The Adafruit BNO 055 was adapted to the surgical handpiece (contra-angle) with which the drilling of the models was performed.

**Fig 2:**
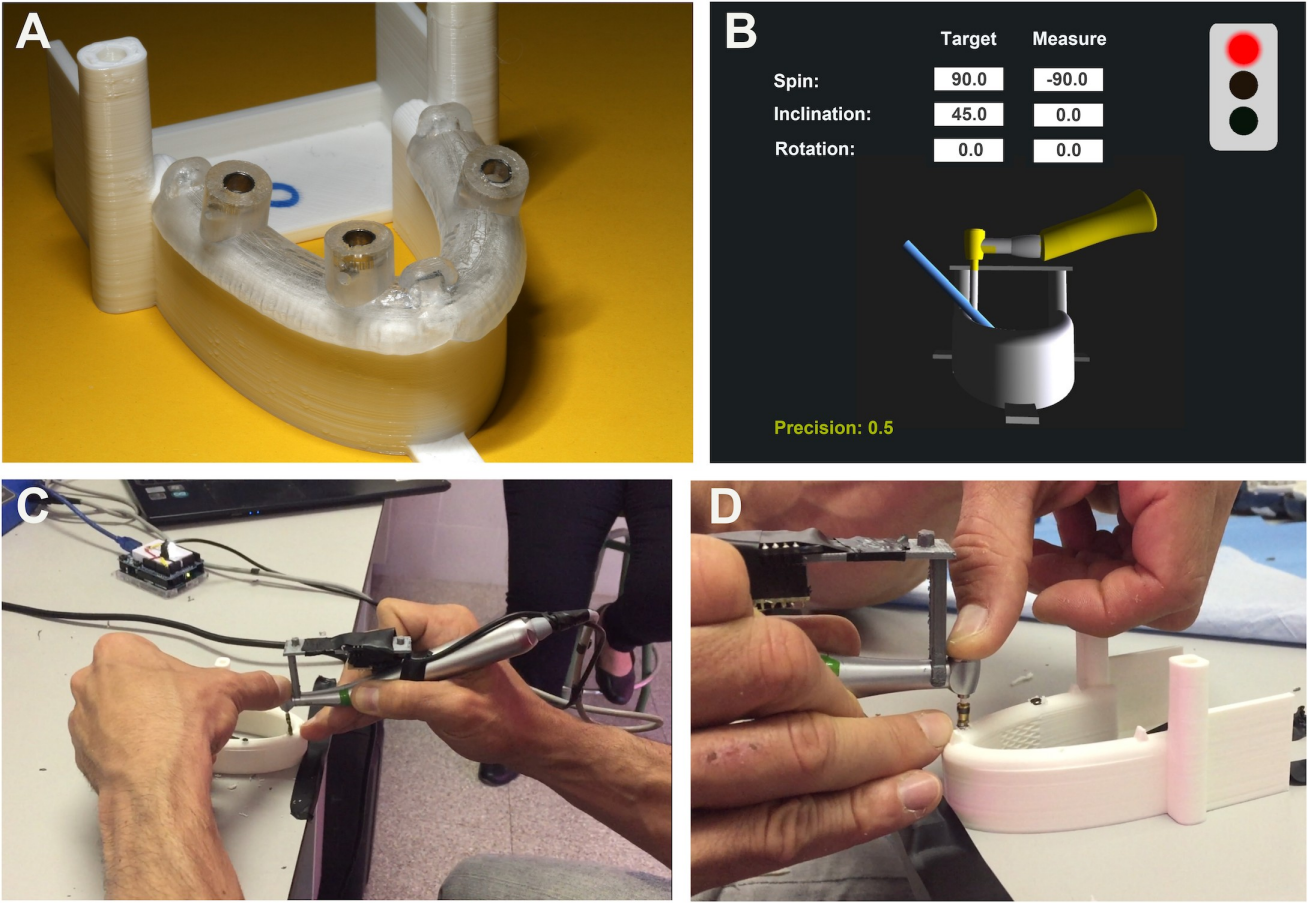
Surgical template and navigator´s graphical interface. (A) Surgical template adapted on the model. (B) Screen of the real time navigation software with IMU adapted to handpiece for drilling the models. The graphical interface includes the location of the handpiece and the drill together with the planned implant axis, enabling the operator to align both indications in real time. (C and D) Pictures of implant placements using the navigator’s graphical interface.

A programmable electronic board based on the microcontroller ATmega328P (Arduino Uno R3) was connect to the Adafruit BNO055 absolute orientation sensor by a serial data bus using the I2C protocol. Specific algorithms for controlling the position of the contra-angle were developed using the Integrated Development Environment Arduino v. 1.8.1 (S4 File). Using this approach, the angulation of the handpiece, and therefore of the drill, can be obtained and easily transferred to the navigator in real time (Fig 2).

The study was performed in accordance with the guidelines and regulations within the training framework of the Master in Implantology programme and approved by the Research Ethics Board of the University Miguel Hernández (Elche, Alicante, Spain). Ten subjects were randomly recruited among teachers and trainees enrolled in this programme. We obtained informed consent of all the subjects before any study procedure was conducted. According to their prior experience in placing implants, the subjects were distributed into two subgroups of 5 persons each: the “experienced operators” (ExO) who had previously placed more than 100 implants in their private practice and the “non-experienced operators” (NExO), who had previously placed less than 20 implants. This second subgroup was composed of recent graduate students enrolled in the programme.

Each subject performed the simulated osteotomy in 2 jaw models, placing 3 implants in each model. The first surgeries were performed using the standard CAD-CAM guiding templates. After finishing the first 3 implants, the subjects had a 30-minute break. Then, they were instructed to use the real-time navigation device to place the remaining 3 implants in the other model.

All the implants were placed the same day, under the same circumstances, and following the same drilling protocol. The drilling sequence used was that recommended by the manufacturer for the Astra Tech EV implant system. For the 4.2 mm wide implant used in this study, drills 1, 3, and 4 were successively used. When drilling through the template, the respective metal sleeves were used for each drill width.

Before each drill, we checked the right alignment of the model as well as all the parameters of the surgical navigation guide and/or the absence of movement of the surgical guide. The reproducibility of the template position was facilitated by the design of the model with 3 triangular protrusions and the total rigidity and absence of mobility of the support.

Finally, a questionnaire was distributed to all participants to assess their opinions regarding (1) feeling of support during drilling, (2) ease of use of the navigation prototype, and (3) general satisfaction with the systems.

**Fig 3:**
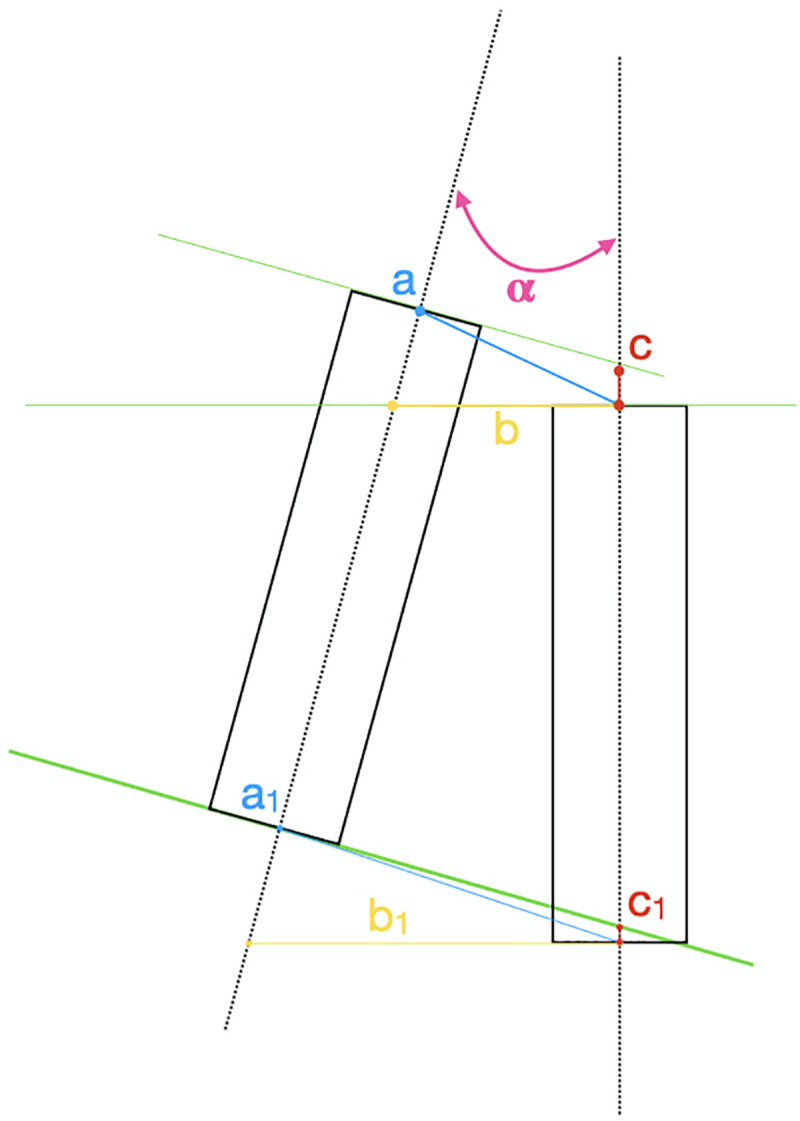
Measures to compare planned with placed implant positions. α: angular deviation (degrees); a: Global deviation at coronal point (mm), b: Lateral deviation at coronal point (mm), c: Depth deviation at coronal point (mm); a1: Global deviation at apical point (mm), b1: Lateral deviation at apical point (mm) c1: Depth deviation at apical point (mm).

All 20 models with the implants placed were scanned with the Planmeca ProMax 3D Plus CBCT imaging unit. Then, DICOM files were imported one by one into Simplant Pro, v 17.01, to obtain the Sprite (SPR) files. Furthermore, an additional SPR file of the initial planning was obtained. The SPR files of the drilled models were superimposed one by one onto the initial planned file. The software Simplant Internal Master (Materialise NV) was used to merge the pre- and postoperative CBCT files using an iterative closest point algorithm (ICP) method. Then, the angular differences between the pre- and postoperative implant positions were compared and automatically analysed by the software. Global, angular, depth and lateral deviation parameters were defined and calculated between the planned and the placed implants, for the apical and coronal positions of the implants (Fig 3).

Statistical analysis was performed using IBM SPSS Software (Version 25.0, IBM Corp.). Data were described with regard to mean values, standard deviations, and 95% confidence intervals. A one-way ANOVA test was used to determine whether there were statistically significant differences between the means of different values. In all cases, *p* < 0.05 was considered statistically significant.

## Results

Sixty implants placed in 20 identical jaw models were included in this study. Each subject placed 3 implants using standard CAD-CAM stereolithographic templates and 3 implants using the novel real-time navigation system. Table 1 shows the mean values for the global, angular, depth, and lateral deviation parameters between the planned and the placed implants for the apical and coronal positions.

**Table 1:** Mean deviations between implant planned and placed positions in the control (SG) and experimental (IMU) groups.

	SG*			IMU**		
	Mean ± SE	SD	95% CI	Mean ± SE	SD	95% CI
**Coronal/Angular**	**5.63 ± 1.41°**	**3.94**	**4.22–7.04**	**7.13 ± 1.47°**	**4.10**	**5.66–8.6**
Coronal/Global	1.42 ± 0.22 mm	0.61	1.2–1.64	1.48 ± 0.21 mm	0.58	1.27–1.69
Coronal/Depth	1.21 ± 0.24 mm	0.66	0.97–1.45	0.98 ± 0.19 mm	0.54	0.78–1.18
Coronal/Lateral	1.00 ± 0.16 mm	0.44	0.84–1.16	0.97 ± 0.21 mm	0.59	0.76–1.18
Apical/Global	2.07 ± 0.35 mm	0.97	1.72–2.42	2.00 ± 0.33 mm	0.93	1.67–2.33
Apical/Depth	1.17 ± 0.22 mm	0.61	0.95–1.39	1.02 ± 0.20 mm	0.57	0.81–1.23
Apical/Lateral	1.54 ± 0.38 mm	1.05	1.16–1.92	1.58 ± 0.36 mm	1.01	1.22–1.94

*SG: Stereolithographic Guide.

**IMU: Inertial Measurement Unit.

Global: Between two points, Coronal (center of the implant platforms), Apical (centers of implant apex), Depth and lateral deviations calculated between the perpendiculars to implant axis.

The primary goal of the study was to comparatively assess the mean angular deviation of the implants from the planned positions related to its final placement for both techniques, stereolithographic guide (SG control group) and novel real-time navigation device (IMU experimental group). The average coronal angle deviation was 5.63 ± 1.41° (SD 3.94; 95% CI, 4.22–7.04) with the surgical template (SG control group) and 7.13 ± 1.47° (SD 4.1; 95% CI, 5.66–8.6) using the navigation prototype (IMU experimental group).

The linear deviations were very similar for implants placed using CAD-CAM templates and the new IMU devices, independently of users’ previous experience (*p* = 0.319). For example, the mean global coronal lateral deviations, between the 2 centre points of the implant platforms were 1.42±0.2 mm and 1.48±0.2 mm for the SG control group and IMU experimental group, respectively. There were also no significant differences in the linear deviations in the apex (*p* = 0.777). Thus, linear deviations in the apex were 2.07±0.35 mm for the SG and 2.00±0.33 mm, for the IMU group.

Table 2 shows the averaged mean angular deviations according to the type of implant for experienced and non-experienced users. No statistically significant differences could be detected between each type of implant for implants placed using standard CAD-CAM stereolithographic templates and implants placed using the novel real-time navigation system based on IMUs (p>0.13). This was valid for both experienced and non-experienced subjects.

**Table 2:** Mean angular deviations in the SG (stereolithographic guide) control group and in the IMU (inertial navigation) test group.

**Stereolitographic Guide**	**IMPLANT 1**	**IMPLANT 2**	**IMPLANT 3**	**ALL IMPLANTS**
**Non-experienced users**	7,17 ± 5,54°	7,20 ± 2,81°	7,07 ± 6,52°	7,15 ± 4,82°
**Experienced users**	3,48 ± 1,93°	4,50 ± 2,61°	4,37 ±1,72°	4,12 ± 2,02°
**IMU**	**IMPLANT 1**	**IMPLANT 2**	**IMPLANT 3**	**ALL IMPLANTS**
**Non-experienced users**	8,97 ± 5,91°	4,96 ± 4,37°	7,98 ± 3,22°	7,30 ± 4,64°
**Experienced users**	3,86 ± 2,88°	8,04 ± 3,59°	8,96 ± 2,62°	6,95 ± 3,65°

Implant 1: 14.74° mesio-distal and -11.89° vestibular-lingual angulations.

Implant 2: 0° angulation in all directions.

Implant 3: 25.29° mesio-distal and -3.85° vestibular-lingual angulations.

On the other hand, and regarding the use of the new IMU device, 70% of the subjects found it highly satisfactory. However, 30% of the subjects considered that previous training was necessary to achieve better results.

## Discussion

The aim of the present work was to evaluate the possible usefulness of IMU sensors to assist surgical navigation to place implants. We compared the precision achieved by the testing inertial navigation device (IMU) with implant placement guided by CAD-CAM templates as a control group (SG).

Because the highest clinical accuracy was reported using the tooth-supported guide [31,32], 3 projections were added to the models to stabilize the support of the guide during the osteotomy, thus simulating a tooth-supported guide. Although significantly greater deviations have been shown in partially guided, compared with fully guided, surgery [33], in the present study, partial guiding was used to provide a better comparison setting between static and dynamic guiding.

The average deviation of the implant-achieved positions using the CAD-CAM guides was found to be in the range of 1 and 2 mm and about 5° of angulation [5,10,12]. In the present experiment, the mean intergroup measures were 6.38° of angular deviation, 1.45 mm of global coronal deviation, and 2.00 mm in the apex, figures consistent with mean data reported in the literature.

However, the average data are not totally relevant; it would be more clinically significant to consider the maximum deviations occasionally reached using the procedure, given the potential complications that could involve. In the present study, the maximum angular deviations were 17.34° (IMU) and 16.68° (SG), and the maximum lateral deviations in the apex were 4.3 mm (IMU) and 4.5 mm (SG), all by a non-experienced operator. Although high, these figures are in line with the reported maximal inaccuracies in the literature. Indeed, 24.9° of deviation in implant angulation [6] and more than 7 mm of deviation in the implant apex [5] have been reported.

The subject’s experience and the learning curve have been considered factors influencing the precision of the guided surgery. Dynamically guided surgery has been shown to require a longer learning curve prior to use than splint surgery [34]. Two in vitro studies using surgical templates showed significantly greater deviations by non-experienced users [35,36]. However, in a similar setting to that of the present study, the subject’s experience was not demonstrated to be a critical factor in achieving the accuracy of implant position [37]. This lack of consistency could be explained by the design of the guide. In the former studies, guides were not fully supported in their distal ends, whereas the latter study used a tooth-supported guide. A similar in-vitro study using fully guiding tooth-supported templates, found only 0.5° more angular deviation for non-experienced users. The authors concluded that these slight differences could be clinically irrelevant [38].

However, there are many other confounding factors potentially influencing these differences, such as the hardness of the resin model, the congruence between sleeves and drills, the position of the model on the bench, or the way the operator holds the guide in place while drilling, to name just a few. With regard to the shape of the jaw models, the present study only focused on evaluating the levels of precision for IMU navigation in vitro and not the implication of anatomical limitations. This is why a simple anatomy has been used without taking into account the detailed characteristics of the jaws after the various situations of edentulism.

Likewise, the placement of the model on a table differs from the real situation in the clinical setting. Despite this, this position allows us a strict correlation with the radiographic acquisition and the planned angulation of the implants.

In future developments of the system, the use of manikin-anchored jaws could be relevant in the approximation of a more realistic environment.

In the present experiment, using a tooth-supported partial guide, non-significant differences between experienced and non-experienced users were found. However, the most deviated values, or outliers, were obtained in the non-experienced users’ group. In this context, it is possible that experience and training may have some influence, at least in preventing the extreme deviations of implant positions.

With the inertial navigation prototype, the perception regarding its ease of use was “satisfactory” for 80% of non-experienced subjects. This qualitative evaluation was consistent with the study by Goodacre et al, where a similar navigation device was valued as “a positive aid*”* to place implants by non-experienced operators [23]. However, these authors only performed a comparison between freehand and inertial dynamic guided drilling. Neither splits were used, nor were implants placed, and the models had a middle line pin as a visual orientation for operators.

In the present study, the inertial navigation device only informed the subjects about the proper angulation of the drilling. The initial point of drilling was determined by a mark in the model, and operators had to match the drill tip with the mark. Although there was no exact transfer of these points, the present study focused mainly on angular deviations. In the clinical setting, the previously planned initial point to drill and the depth of the implant are 2 easily reproducible factors. Given that dynamic navigation is an open process, not blinded like a surgical template, the implantation area is directly visualized, and these 2 planned aspects of the implant position can be easily decided at the time of surgery [39]. By contrast, the osteotomy to place an implant must be adapted to the different angulations of the anatomy. Therefore, the tridimensional angulation of drilling could be considered highly relevant and the factor that will determine the clinical outcome to a greater extent [11], provided that the initial point of insertion and the final depth were properly achieved.

With regard to costs, in all cases, guided surgery presents an added cost. Static guiding involves the cost of manufacturing the CAD-CAM stent. Some authors described different analogical methods trying to reduce the manufacturing cost of the guide, but all should still be scientifically validated. Currently, the cost of the guide can be definitely lowered by the generalization of the 3D printing technology, paving the way to design and manufacture the templates directly in the dental office [40–42]. On the other hand, in dynamic navigation, cost derives from the acquisition of the equipment and its software that, in the case of optical navigation, can be as high as several tens of thousands of euros [43]. Furthermore, an additional cost of about 50 euros per patient should be added for manufacturing the individual plates with the fiduciary markers [44]. Such higher costs are perhaps the main limitation for an extensive use of dynamic navigation guided-surgery. Generally speaking, the additional costs of guided-surgery procedures may exceed the patient’s financial resources [45].

It might be thought that simplifying the guidance system, while maintaining its precision, would facilitate its wide acceptance by clinicians. Thus, the better implant positioning obtained with guided-surgery would result in fewer complications for patients on a larger scale. Undoubtedly, future research in the scope of computer-assisted implant surgery will aim to reduce the costs and increase the simplicity of the systems. This is the final objective of testing the potential clinical application of inertial navigation in the present study. The prototype device has been shown to be feasible to place implants with precision similar to the stereolithographic guides, the current standard. The new system can be simpler, less expensive, and easier to use, provided that the interface can be improved and the operator respects the necessary learning curve of use.

In conclusion, this proof-to-concept study did not find significant differences between test and control groups. Therefore, the precision of the novel surgical navigation tested in the study, based on IMUs, can be said to be comparable to the precision data from the current gold-standard guiding system. Within its limitations, the present study opened the way to consider surgical navigation based on IMUs as a potential tool to guide implant placement. However, more studies are still needed to translate this new approach from an in vitro setting into clinical practice.

## Supporting information

S1 File Descriptive data and distribution box plot charts. (PDF)

S2 File Analysis of variance (ANOVA). (PDF)

S3 File Median charts. (PDF)

S4 File Arduino programming. (PDF)
