# Supplementary material for: A new inertial navigation system for guiding implant placement. An in-vitro proof-of-concept study
Source: PLoS One. 2021 Oct 21;16(10):e0255481. doi: 10.1371/journal.pone.0255481 (PMC8530356; doi:10.1371/journal.pone.0255481)

Descriptive Data (Angular Deviation)

SURGICAL GUIDE		ImplanteNumber					
		1,00		2,00		3,00	
		Experience		Experience		Experience	
		NExO	ExO	NExO	ExO	NExO	ExO
Coronal Angular	Mean	7,17	3,48	7,20	4,50	7,07	4,37
	Maximum	16,68	6,51	10,82	7,94	15,50	6,60
	Median	6,21	3,26	5,73	3,36	2,57	4,34
	Minimum	3,04	1,70	4,35	1,71	2,11	2,31
	Mode	3,04	1,70	4,35	1,71	2,11	2,31
	Standard error	2,48	,86	1,25	1,17	2,92	,77
	Standar deviation	5,54	1,93	2,81	2,61	6,52	1,72
	Range	13,65	4,81	6,47	6,22	13,39	4,29
	Count	5	5	5	5	5	5

IMU		ImplanteNumber					
		1,00		2,00		3,00	
		Experience		Experience		Experience	
		NExO	ExO	NExO	ExO	NExO	ExO
Coronal Angular	Mean	8,97	3,86	4,96	8,04	7,98	8,96
	Maximum	17,34	8,43	11,80	12,47	12,10	13,08
	Median	7,26	3,84	5,35	6,90	7,41	8,82
	Minimum	2,28	1,31	,95	3,94	4,59	6,24
	Mode	2,28	1,31	,95	3,94	4,59	6,24
	Standard error	2,64	1,29	1,95	1,61	1,44	1,17
	Standar deviation	5,91	2,88	4,37	3,60	3,22	2,62
	Range	15,07	7,12	10,85	8,52	7,51	6,84
	Count	5	5	5	5	5	5

Non-experienced users: Box-Plot (all)

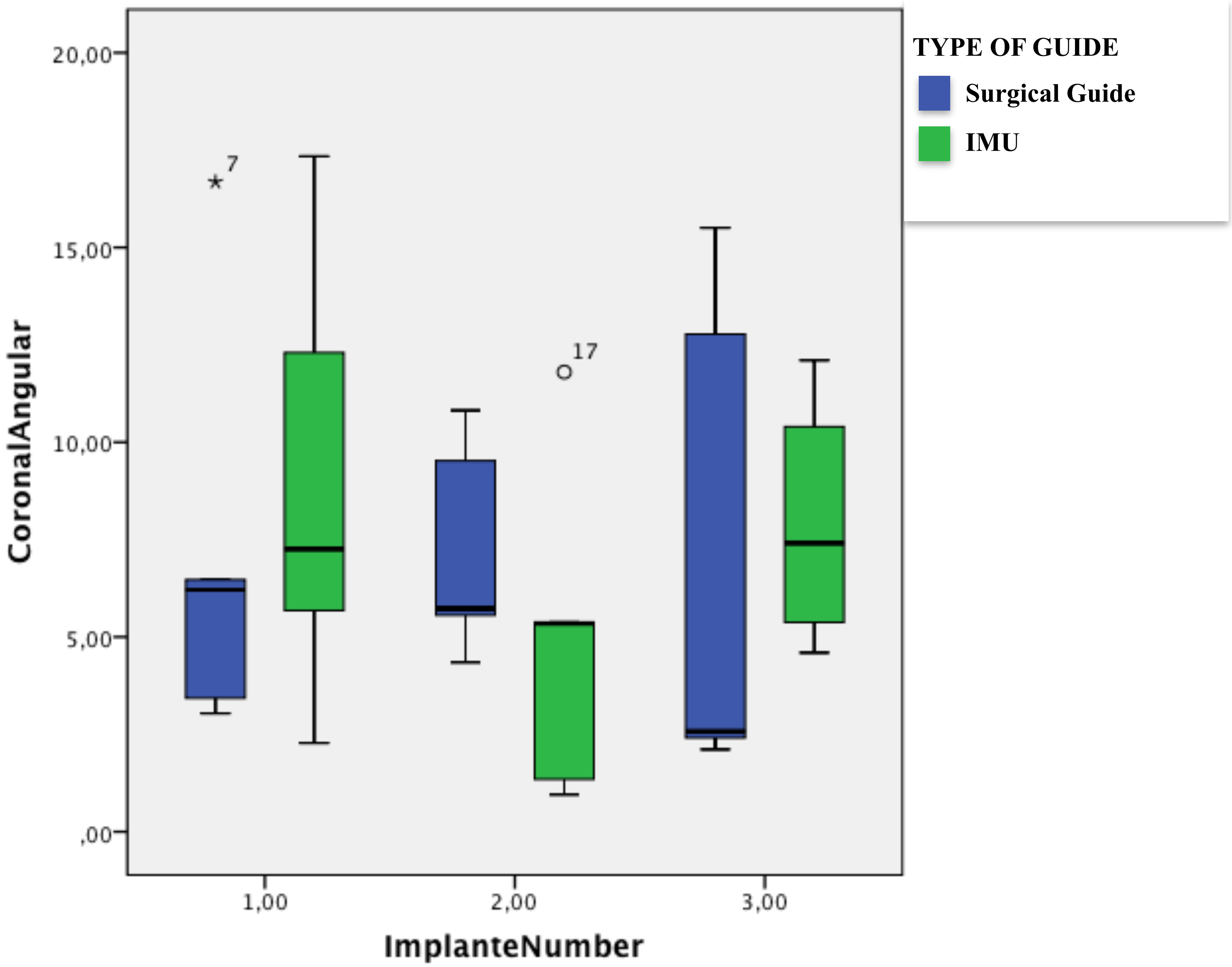

Non-experienced users: Distribution (all)

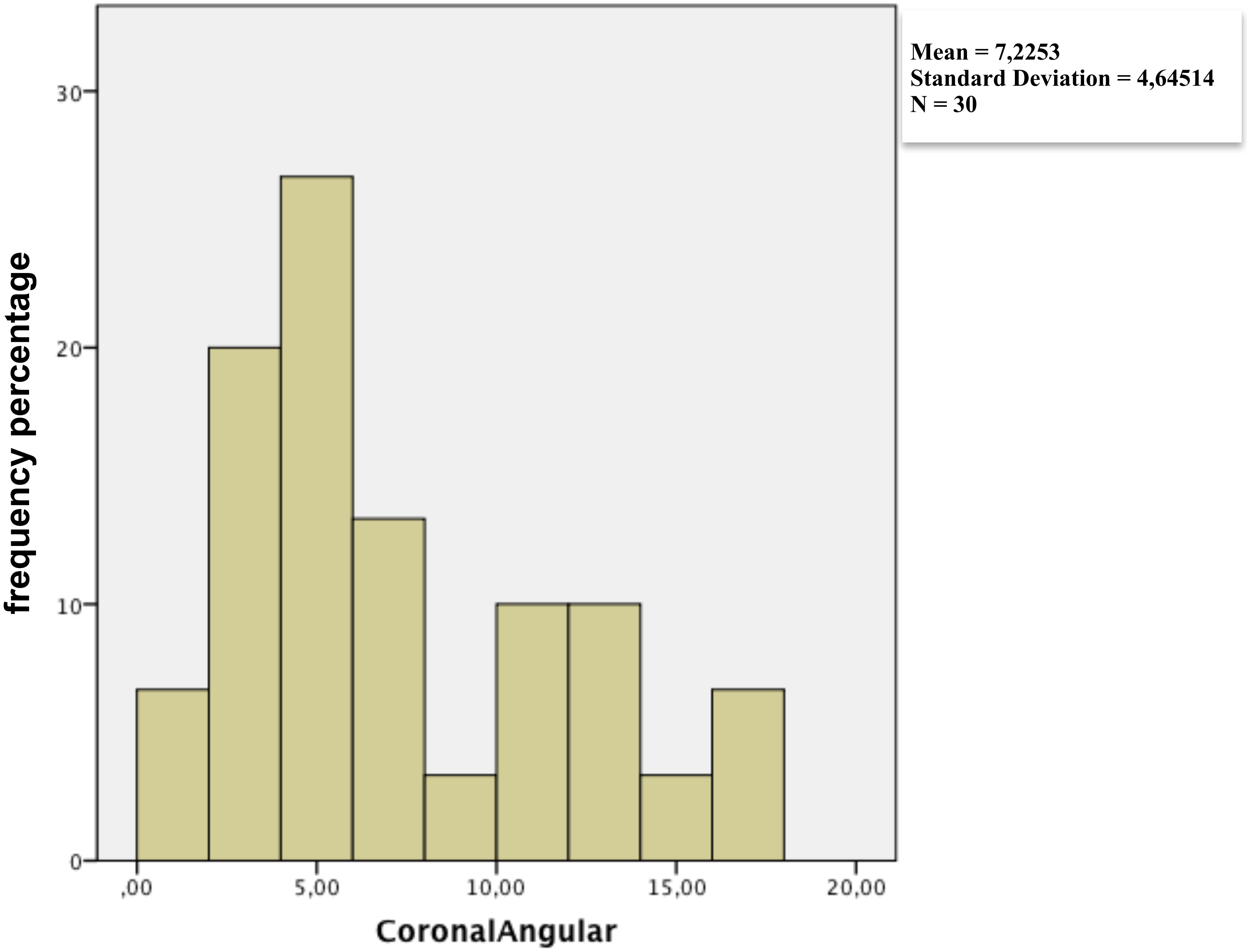

Experienced users: Box-Plot (all)

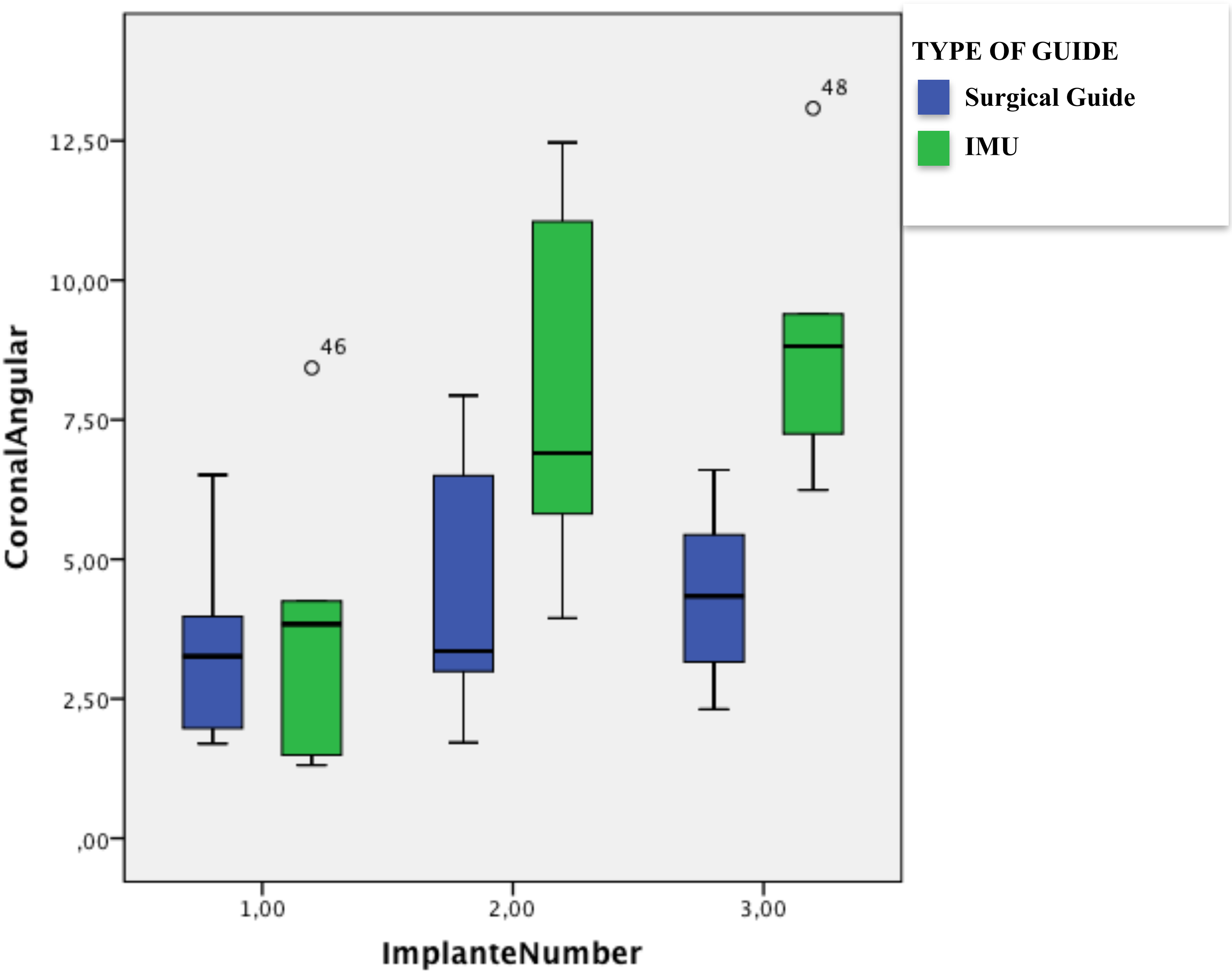

Experienced users: Distribution (all)

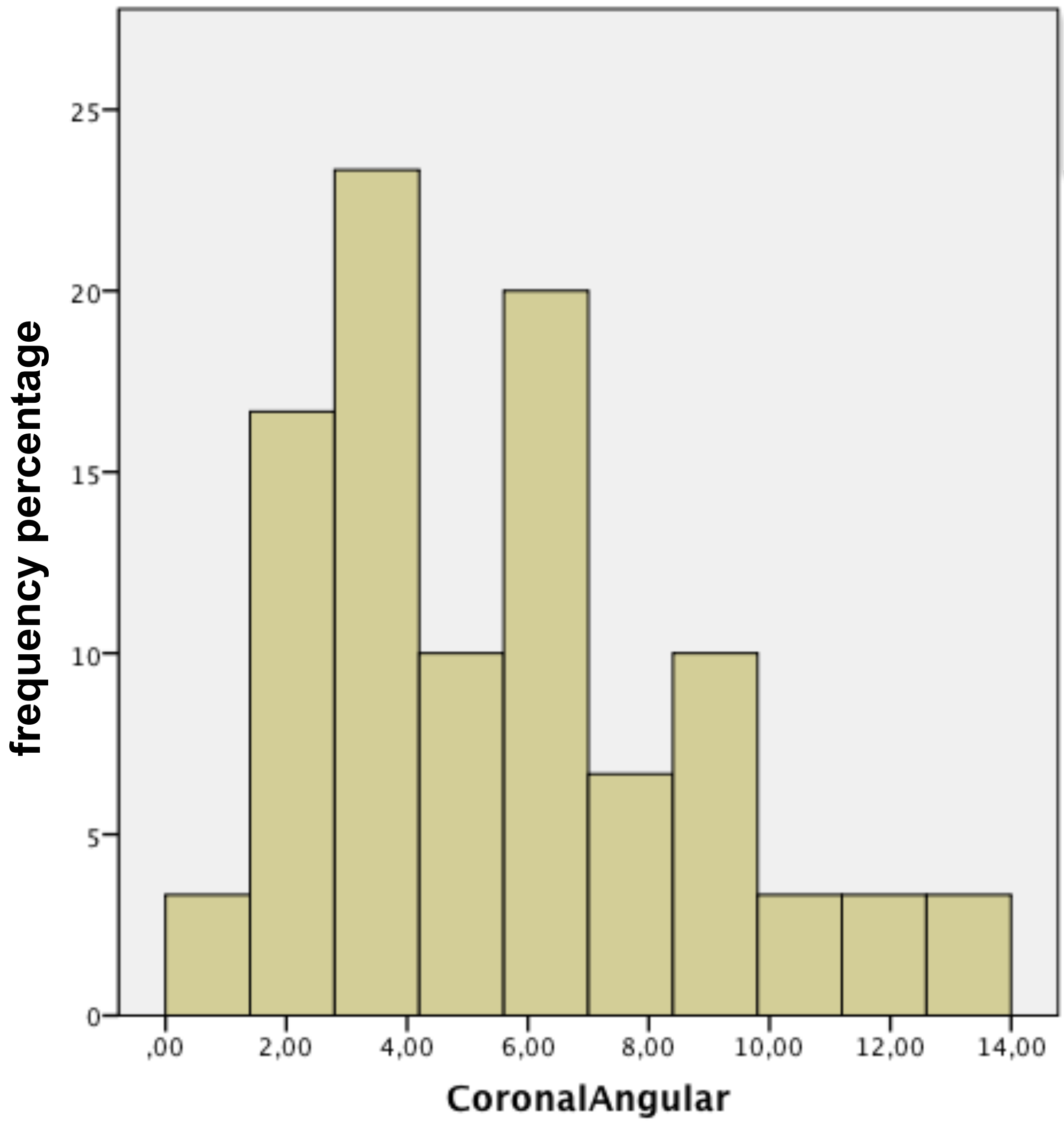

Mean = 5,5351
Standard Deviation = 3,23372
N = 30

Angled Implants distribution (= 1 y 3) with SG: all users

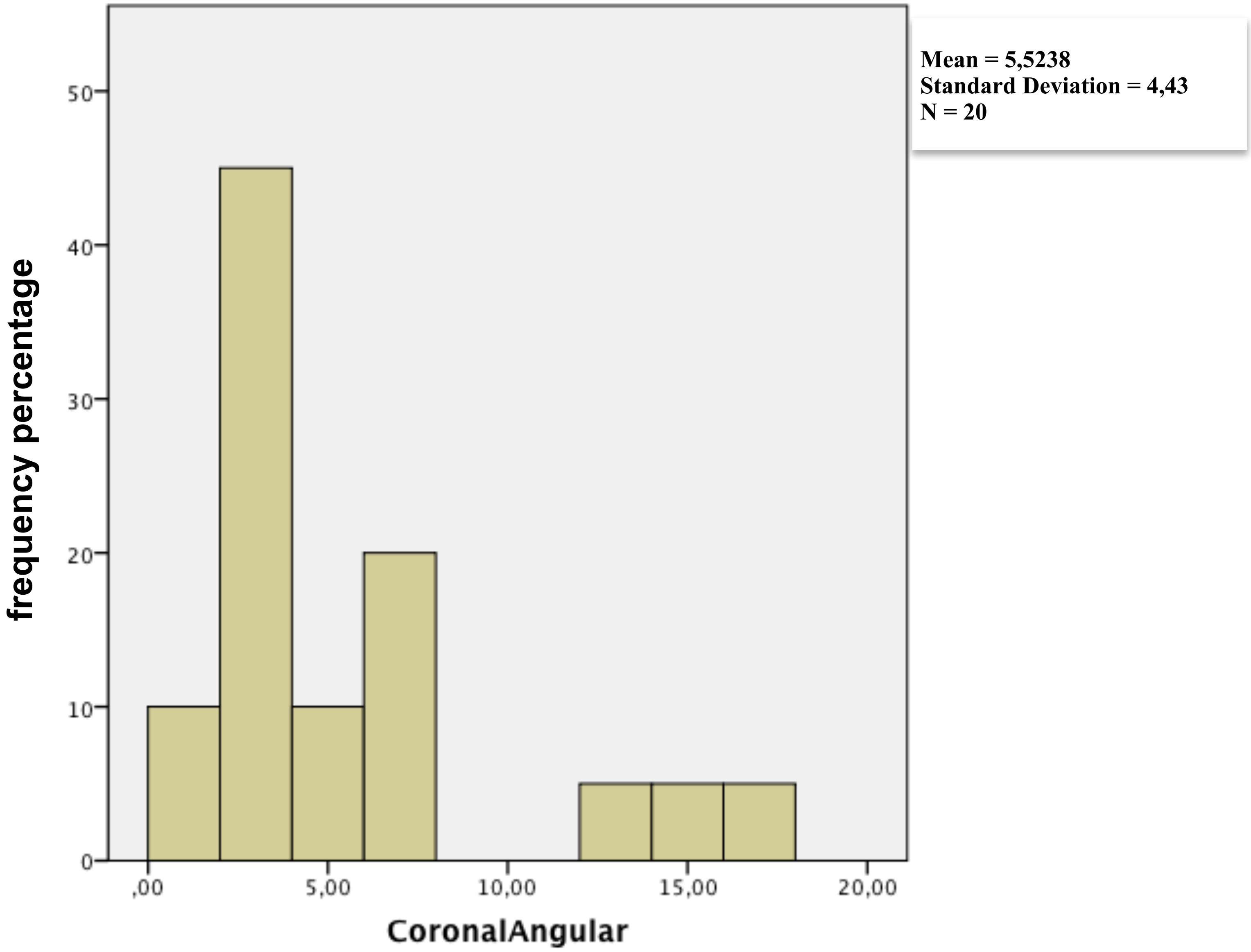

Non angled implants distribution (= 2) with SG: all users

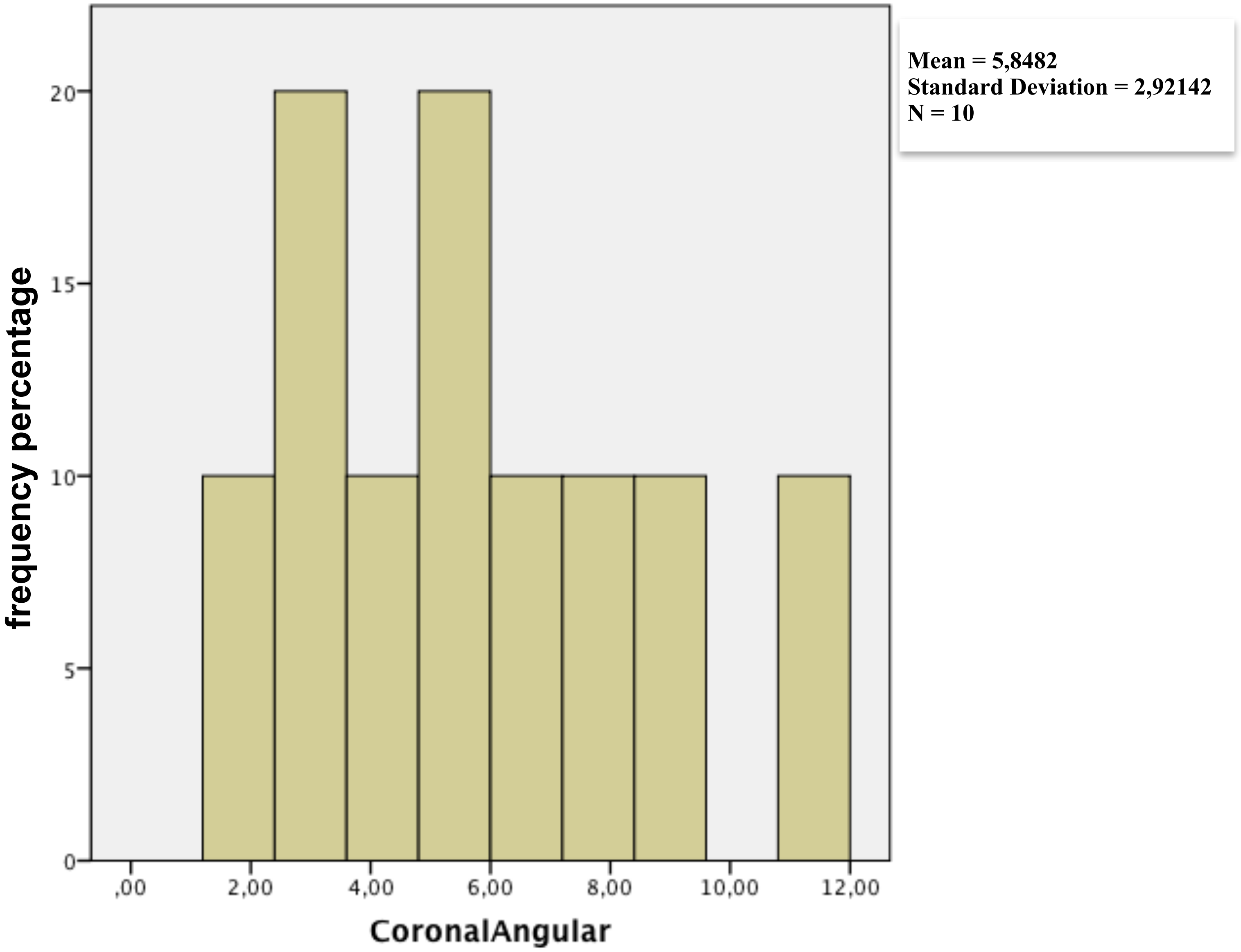

Angled Implants distribution (= 1 y 3) with IMU: all users

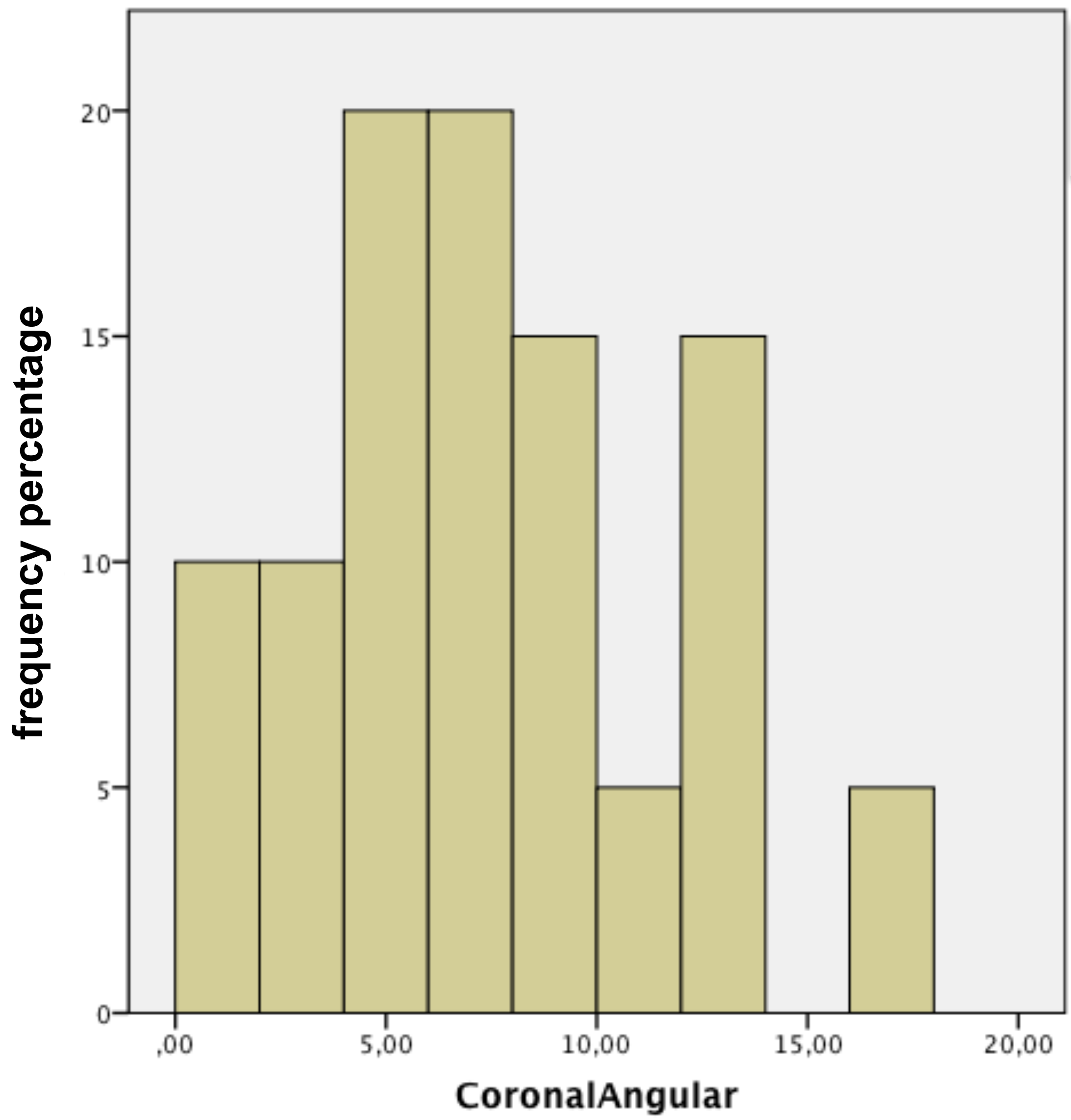

Mean = 7,4424
Standard Deviation = 4,17019
N = 20

Non angled implants distribution (= 2) with IMU: all users

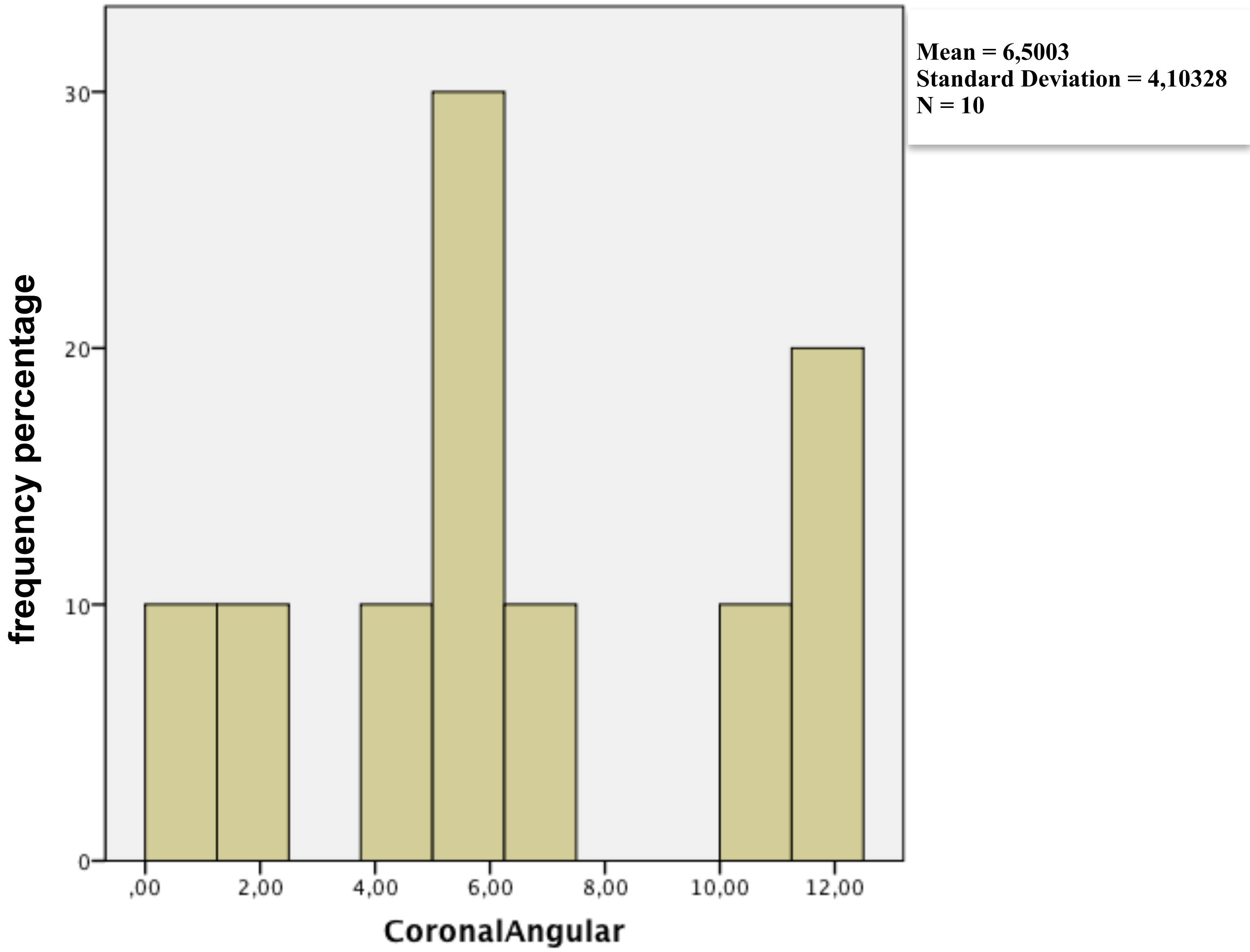

Supplement: S1 File — (PDF) [file pone.0255481.s001.pdf]
