# Supplementary material for: A new inertial navigation system for guiding implant placement. An in-vitro proof-of-concept study
Source: PLoS One. 2021 Oct 21;16(10):e0255481. doi: 10.1371/journal.pone.0255481 (PMC8530356; doi:10.1371/journal.pone.0255481)
Supplement: S2 File — (PDF) [file pone.0255481.s002.pdf]

Non Experienced Users

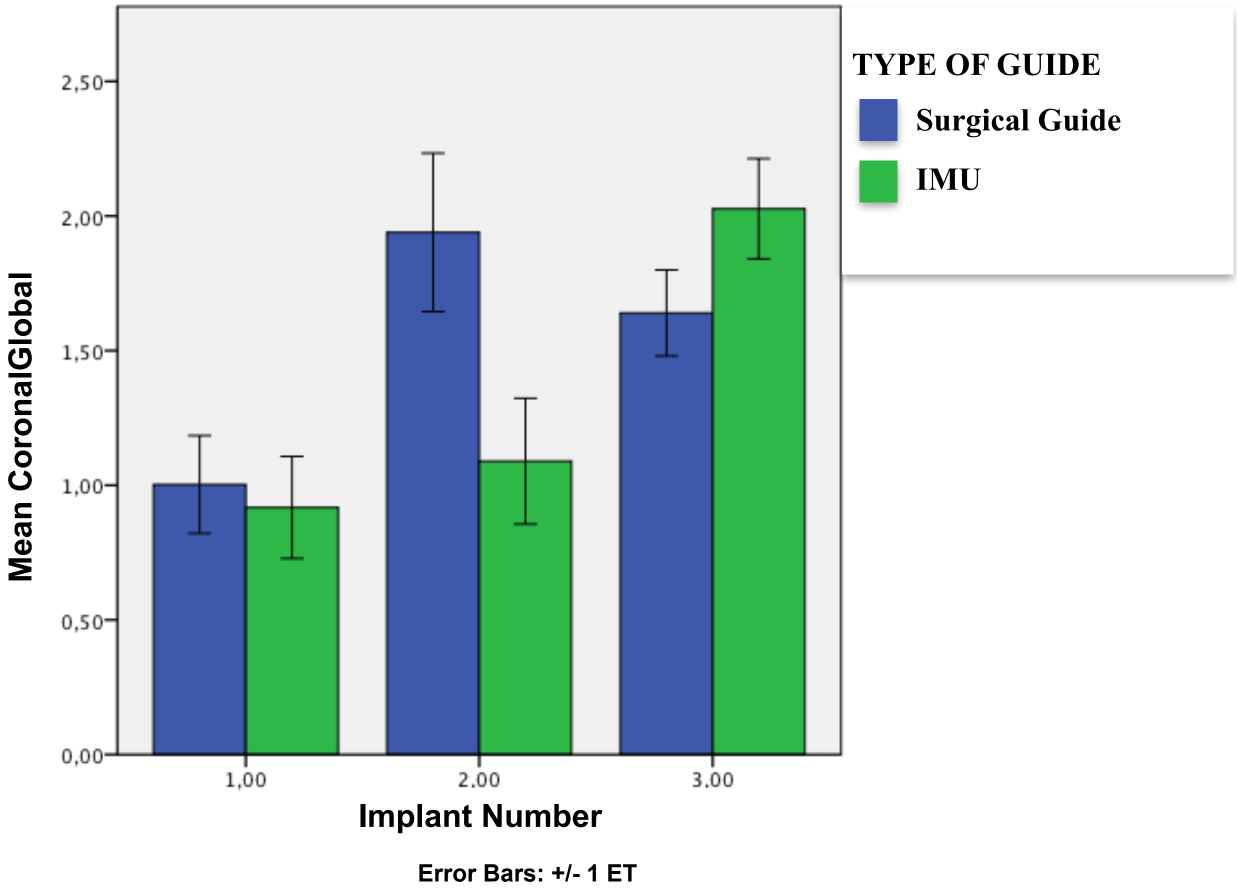

Non Experienced Users

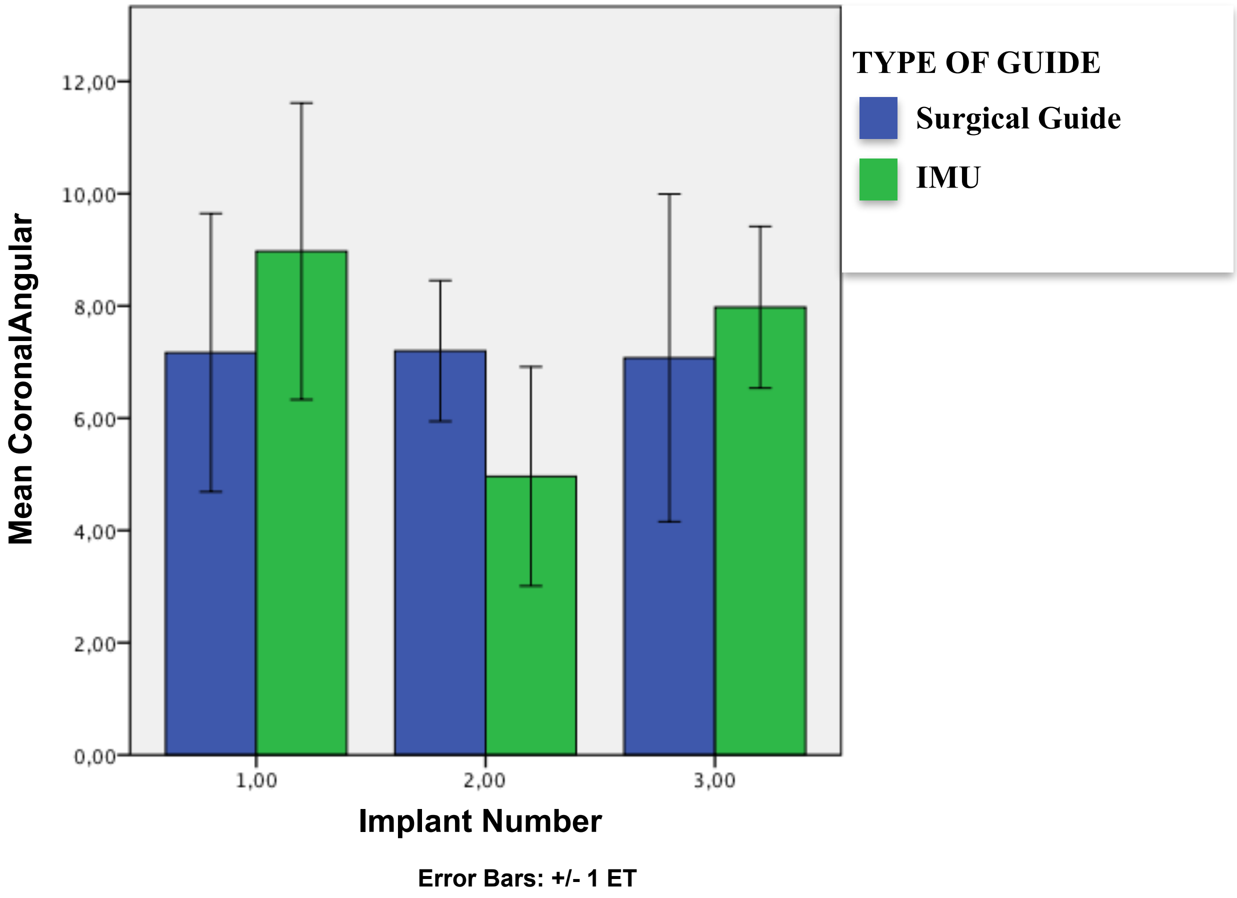

Non Experienced Users

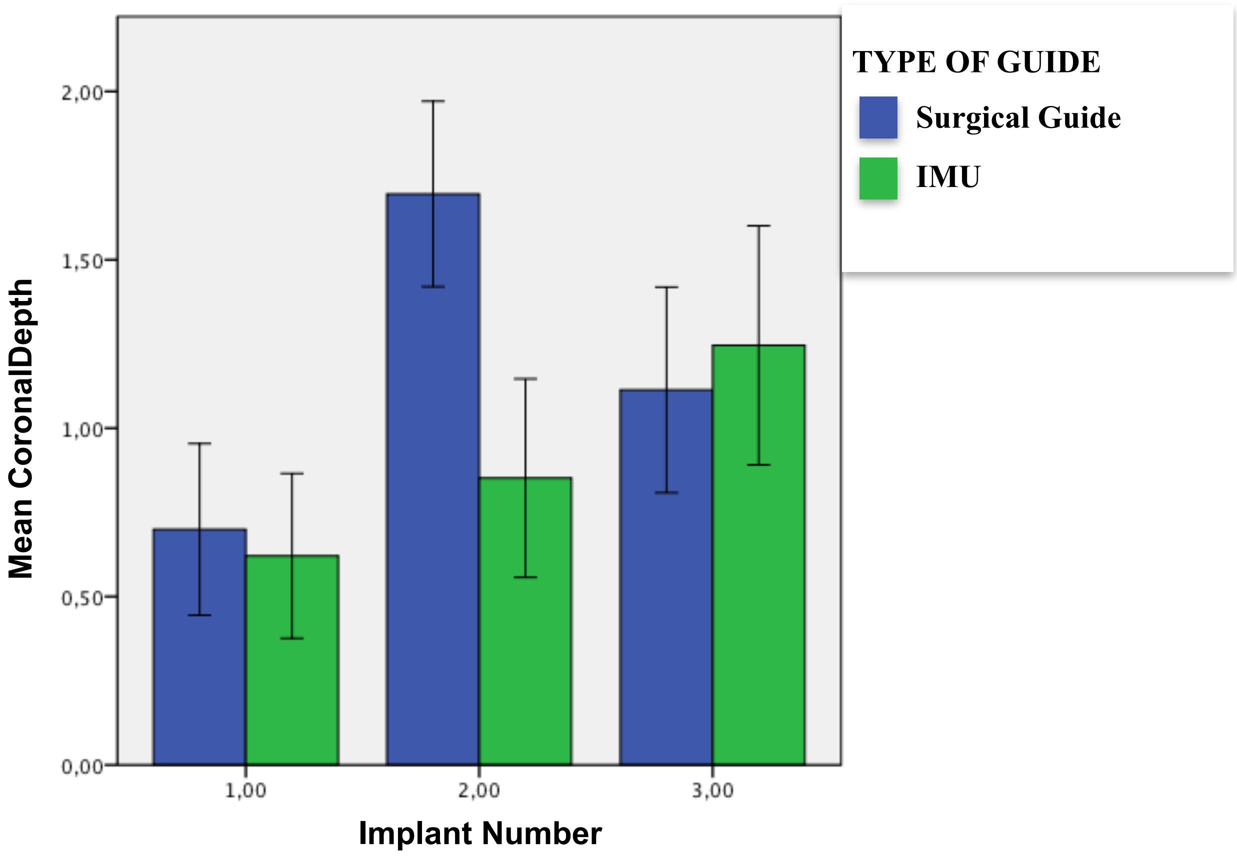

Error Bars: +/- 1 ET

Non experienced Users

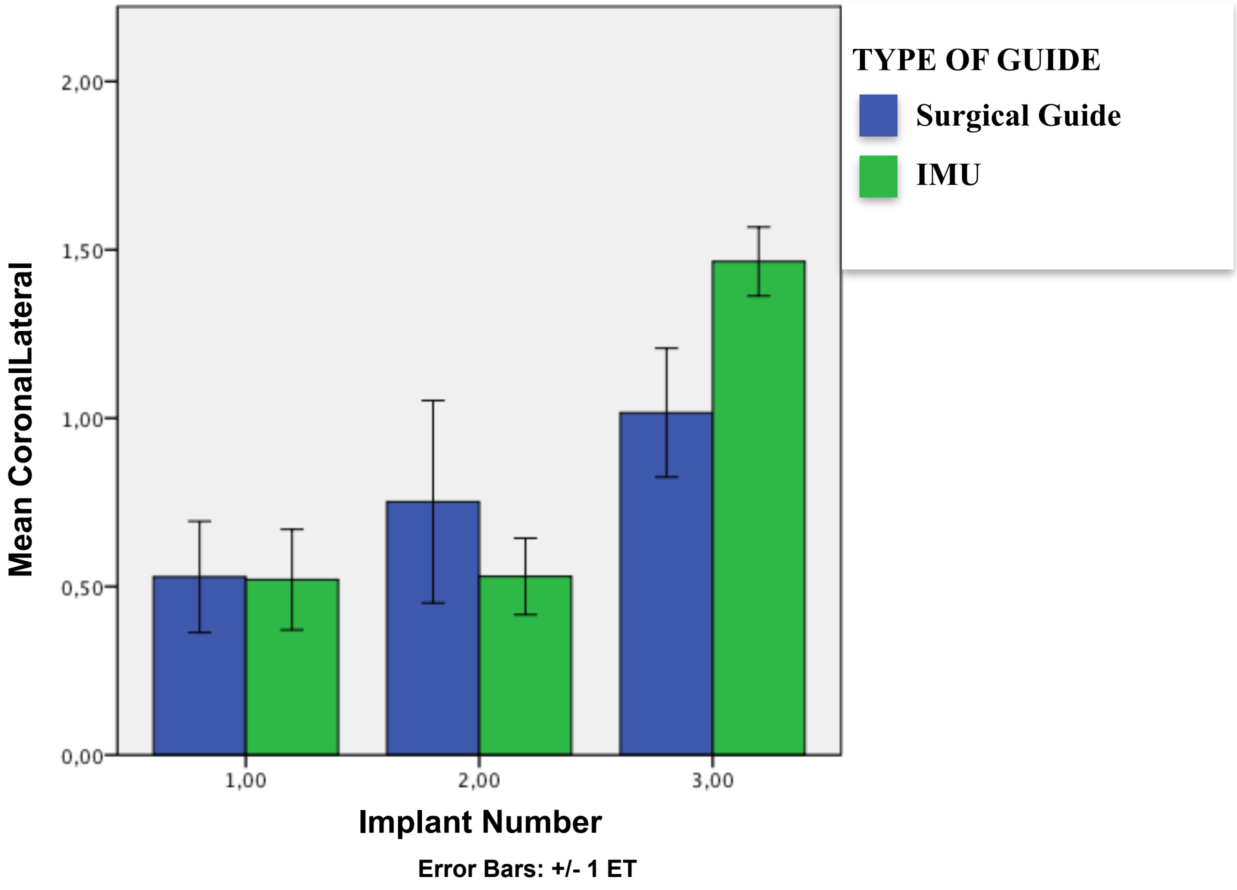

Experienced Users

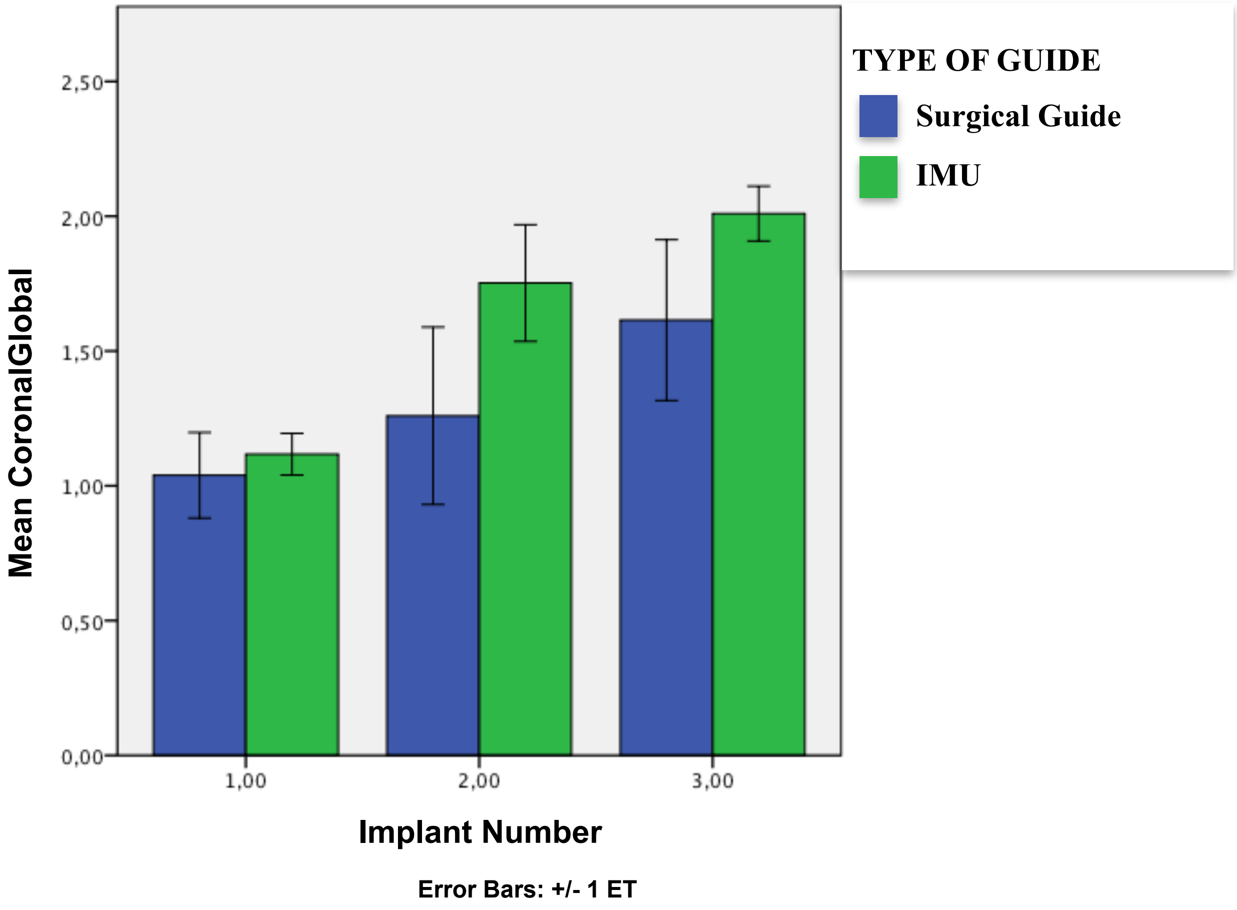

Experienced Users

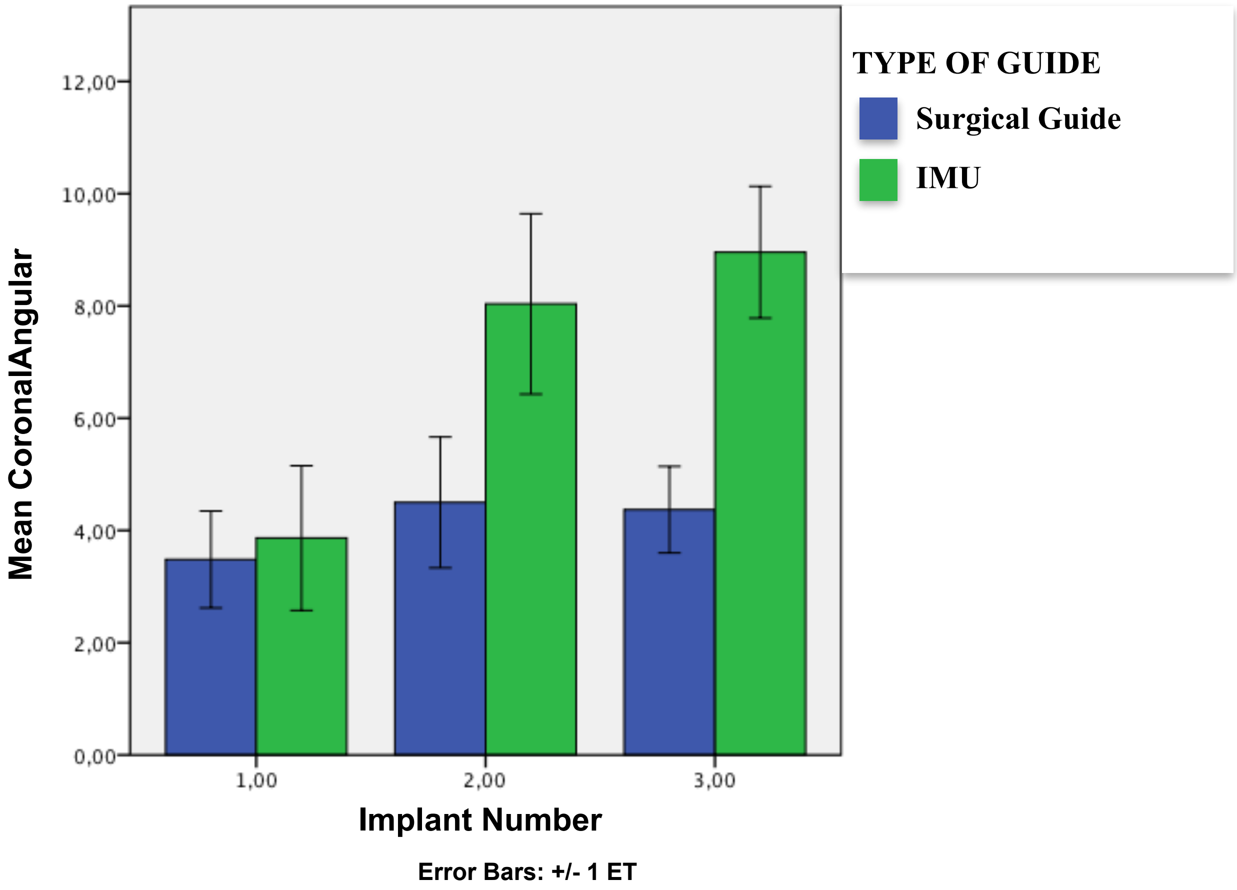

Experienced Users

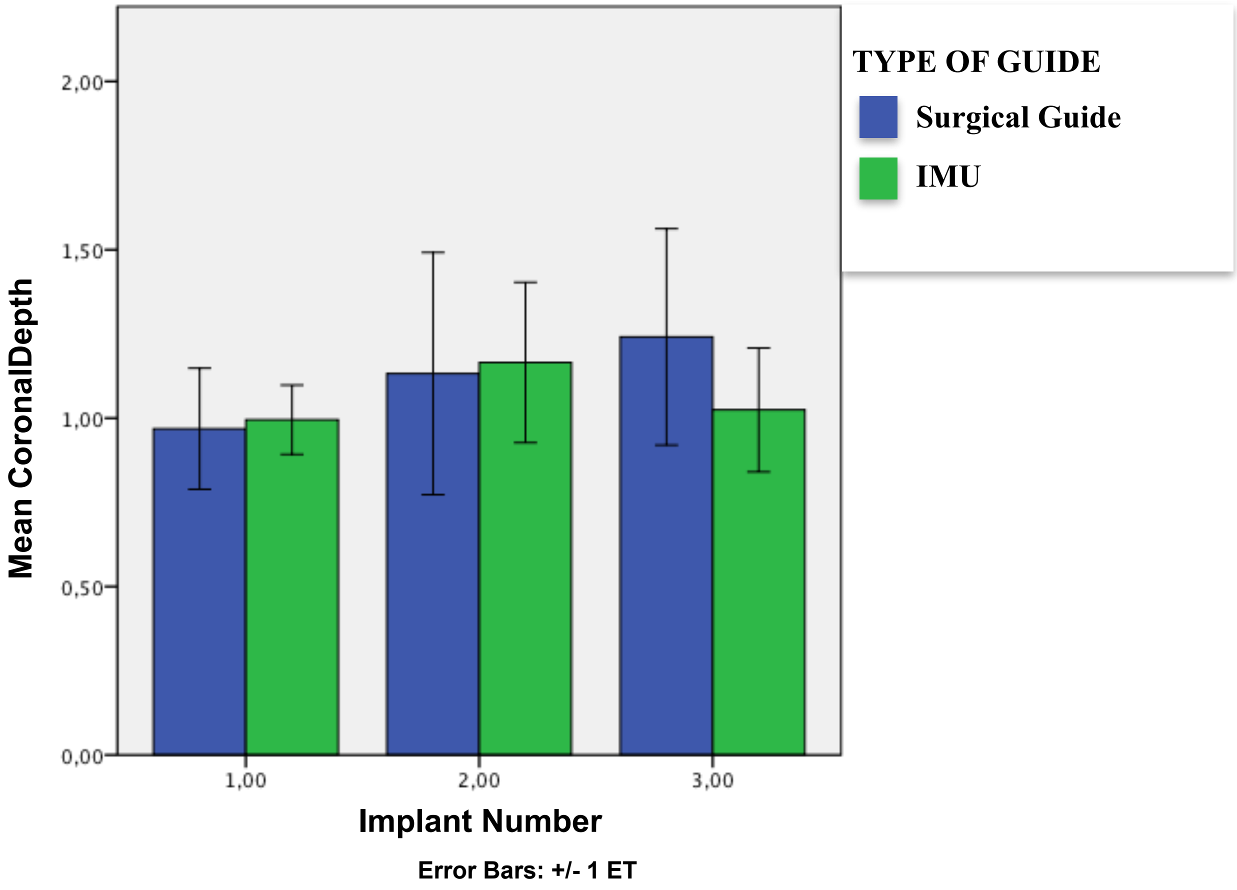

Experienced Users

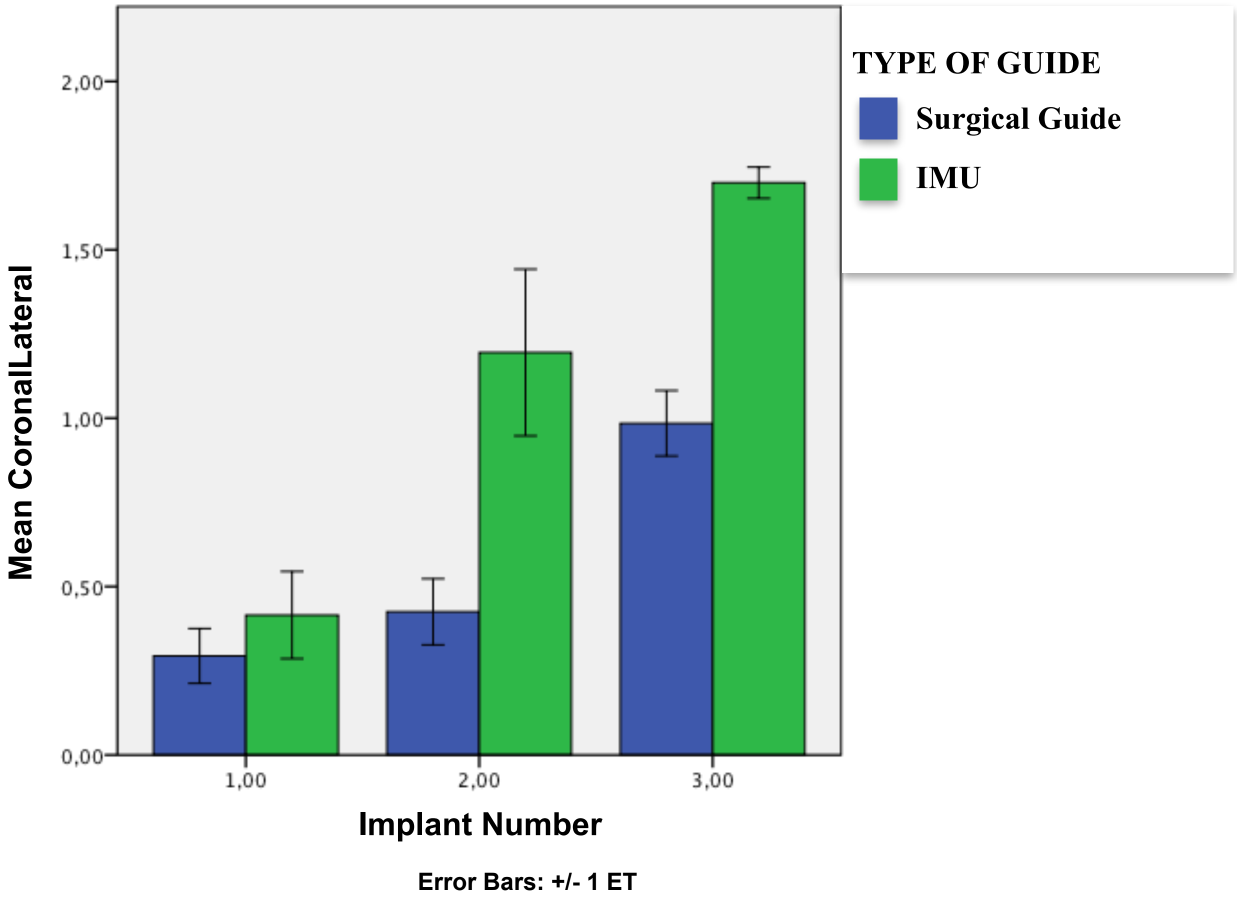

ANOVA ALL USERS

(if there are significant statistical differences between the use of the Stereolithographic Splint and the new device in the different measurements.)

ONE-FACTOR ANOVA

		Sum of square	gl	Root Mean Square	F	p
CoronalGlobal	Inter-groups	,073	1	,073	,202	,655
	Intra-groups	20,892	58	,360		
	Total	20,965	59			
CoronalAngular	Inter-groups	33,592	1	33,592	2,077	,155
	Intra-groups	938,255	58	16,177		
	Total	971,847	59			
CoronalDepth	Inter-groups	,374	1	,374	1,011	,319
	Intra-groups	21,440	58	,370		
	Total	21,814	59			
CoronalLateral	Inter-groups	1,387	1	1,387	5,028	,029
	Intra-groups	16,004	58	,276		
	Total	17,391	59			
ApicalGlobal	Inter-groups	,074	1	,074	,081	,777
	Intra-groups	53,228	58	,918		
	Total	53,302	59			
ApicalDepth	Inter-groups	,340	1	,340	,952	,333
	Intra-groups	20,699	58	,357		
	Total	21,039	59			
ApicalLateral	Inter-groups	,021	1	,021	,020	,888
	Intra-groups	62,190	58	1,072		
	Total	62,211	59			

Only statistically significant ($P<0.05$): Coronal Lateral

ANOVA NON EXPERIENCED USERS

(if there are significant statistical differences between the use of the Stereolithographic Splint and the new device in the different measurements.)

ONE-FACTOR ANOVA

		Sum of square	gl	Root Mean Square	F	p
CoronalGlobal	Inter-groups	,251	1	,251	,623	,437
	Intra-groups	11,256	28	,402		
	Total	11,507	29			
CoronalAngular	Inter-groups	,188	1	,188	,008	,928
	Intra-groups	625,555	28	22,341		
	Total	625,743	29			
CoronalDepth	Inter-groups	,520	1	,520	1,066	,311
	Intra-groups	13,642	28	,487		
	Total	14,161	29			
CoronalLateral	Inter-groups	,040	1	,040	,149	,702
	Intra-groups	7,541	28	,269		
	Total	7,581	29			
ApicalGlobal	Inter-groups	2,326	1	2,326	2,385	,134
	Intra-groups	27,300	28	,975		
	Total	29,626	29			
ApicalDepth	Inter-groups	,657	1	,657	1,567	,221
	Intra-groups	11,742	28	,419		
	Total	12,399	29			
ApicalLateral	Inter-groups	1,829	1	1,829	1,432	,241
	Intra-groups	35,763	28	1,277		
	Total	37,592	29			

No difference is statistically significant.

ANOVA EXPERIENCED USERS

(if there are significant statistical differences between the use of the Stereolithographic Splint and the new device in the different measurements.)

ONE-FACTOR ANOVA

		Sum of square	gl	Root Mean Square	F	p
CoronalGlobal	Inter-groups	,778	1	,778	2,514	,124
	Intra-groups	8,667	28	,310		
	Total	9,445	29			
CoronalAngular	Inter-groups	60,259	1	60,259	6,944	,014
	Intra-groups	242,992	28	8,678		
	Total	303,251	29			
CoronalDepth	Inter-groups	,021	1	,021	,076	,785
	Intra-groups	7,594	28	,271		
	Total	7,615	29			
CoronalLateral	Inter-groups	2,148	1	2,148	7,864	,009
	Intra-groups	7,646	28	,273		
	Total	9,794	29			
ApicalGlobal	Inter-groups	1,299	1	1,299	1,771	,194
	Intra-groups	20,534	28	,733		
	Total	21,833	29			
ApicalDepth	Inter-groups	,000	1	,000	,001	,981
	Intra-groups	8,533	28	,305		
	Total	8,533	29			
ApicalLateral	Inter-groups	2,431	1	2,431	3,429	,075
	Intra-groups	19,854	28	,709		
	Total	22,285	29			

Only statistically significant ($P<0.05$): CoronalAngular and CoronalLateral
