# Supplementary material for: A new inertial navigation system for guiding implant placement. An in-vitro proof-of-concept study
Source: PLoS One. 2021 Oct 21;16(10):e0255481. doi: 10.1371/journal.pone.0255481 (PMC8530356; doi:10.1371/journal.pone.0255481)
Supplement: S3 File — (PDF) [file pone.0255481.s003.pdf]

All users

	ImplantNumber											
	1,00				2,00				3,00			
	TypeGuide				TypeGuide				TypeGuide			
	Surgical Guide		IMU		Surgical Guide		IMU		Surgical Guide		IMU	
	Mean	Std Error of Mean	Mean	Std Error of Mean	Mean	Std Error of Mean	Mean	Std Error of Mean	Mean	Std Error of Mean	Mean	Std Error of Mean
CoronalGlobal	1,02	,11	1,02	,10	1,60	,24	1,42	,19	1,63	,16	2,02	,10
CoronalAngular	5,33	1,38	6,42	1,63	5,85	,92	6,50	1,30	5,72	1,49	8,47	,89
CoronalDepth	,83	,15	,81	,14	1,41	,23	1,01	,19	1,18	,21	1,14	,19
CoronalLateral	,41	,10	,47	,09	,59	,16	,86	,17	1,00	,10	1,58	,07
ApicalGlobal	1,81	,27	1,88	,31	2,35	,33	2,34	,36	2,07	,33	1,79	,20
ApicalDepth	,87	,14	,83	,14	1,46	,22	1,11	,21	1,19	,19	1,13	,19
ApicalLateral	1,39	,34	1,57	,35	1,69	,34	1,98	,35	1,56	,35	1,21	,23

Non Experienced Users (1-5)

	ImplantNumber											
	1,00				2,00				3,00			
	TypeGuide				TypeGuide				TypeGuide			
	Surgical Guide		IMU		Surgical Guide		IMU		Surgical Guide		IMU	
	Mean	Std Error of Mean	Mean	Std Error of Mean	Mean	Std Error of Mean	Mean	Std Error of Mean	Mean	Std Error of Mean	Mean	Std Error of Mean
CoronalGlobal	1,00	,18	,92	,19	1,94	,29	1,09	,23	1,64	,16	2,03	,19
CoronalAngular	7,17	2,48	8,97	2,64	7,20	1,25	4,96	1,95	7,07	2,92	7,98	1,44
CoronalDepth	,70	,25	,62	,24	1,70	,28	,85	,29	1,11	,31	1,25	,36
CoronalLateral	,53	,17	,52	,15	,75	,30	,53	,11	1,02	,19	1,47	,10
ApicalGlobal	2,21	,49	2,22	,55	2,86	,47	1,79	,48	2,40	,41	1,79	,33
ApicalDepth	,78	,23	,63	,22	1,74	,22	,93	,33	1,09	,21	1,16	,36
ApicalLateral	1,95	,55	2,05	,58	2,10	,61	1,41	,46	1,98	,54	1,09	,38

Experienced users (6-10)

	ImplantNumber											
	1,00				2,00				3,00			
	TypeGuide				TypeGuide				TypeGuide			
	Surgical Guide		IMU		Surgical Guide		IMU		Surgical Guide		IMU	
	Mean	Std Error of Mean	Mean	Std Error of Mean	Mean	Std Error of Mean	Mean	Std Error of Mean	Mean	Std Error of Mean	Mean	Std Error of Mean
CoronalGlobal	1,04	,16	1,12	,08	1,26	,33	1,75	,22	1,61	,30	2,01	,10
CoronalAngular	3,48	,86	3,86	1,29	4,50	1,17	8,04	1,61	4,37	,77	8,96	1,17
CoronalDepth	,97	,18	1,00	,10	1,13	,36	1,17	,24	1,24	,32	1,02	,18
CoronalLateral	,29	,08	,42	,13	,43	,10	1,20	,25	,98	,10	1,70	,05
ApicalGlobal	1,40	,11	1,54	,27	1,83	,37	2,89	,44	1,74	,51	1,78	,25
ApicalDepth	,97	,20	1,02	,12	1,18	,36	1,30	,28	1,28	,33	1,09	,18
ApicalLateral	,82	,25	1,10	,30	1,28	,28	2,54	,41	1,15	,41	1,32	,30
