# Supplementary material for: A new inertial navigation system for guiding implant placement. An in-vitro proof-of-concept study
Source: PLoS One. 2021 Oct 21;16(10):e0255481. doi: 10.1371/journal.pone.0255481 (PMC8530356; doi:10.1371/journal.pone.0255481)

Arduino programming.

By creating the corresponding electrical connections, the programming of the Arduino development board can proceed. In the following figure it can be seen that the power supply to the Adafruit BNO 055 unit is provided by the red wire (+5v) and the black wire (GND) of the Arduino board. The yellow (SDA) and green (SCL) wires create the I2C connection between the Arduino and the Adafruit BNO 055 unit. Electrical connections on Arduino Board can be seen from descubrearduino.com.

The programming of the Arduino development board is done using the official Arduino software in version 1.8.1: an integrated development environment IDE (Integrated Development Environment). The program, developed in the C language for Arduino, complies with the following block diagram:

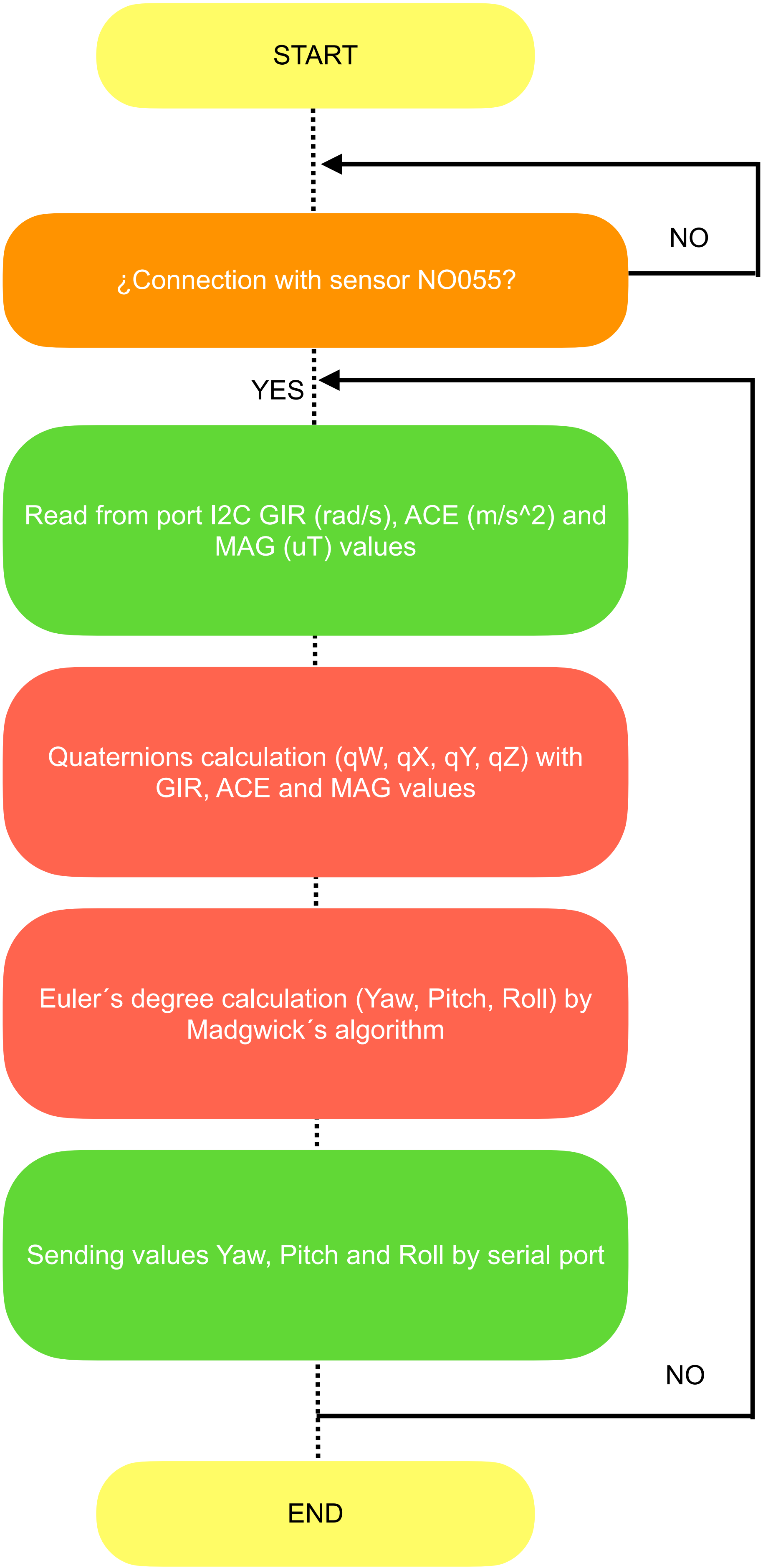
START
¿Connection with sensor NO055?
NO
YES
Read from port I2C GIR (rad/s), ACE (m/s^2) and MAG (uT) values
Quaternions calculation (qW, qX, qY, qZ) with GIR, ACE and MAG values
Euler´s degree calculation (Yaw, Pitch, Roll) by Madgwick´s algorithm
Sending values Yaw, Pitch and Roll by serial port
NO
END

The program was installed on a computer with Ubuntu operating system version 16.04 and using a USB cable connecting the Arduino development board and the computer with Ubuntu as the operating system.

For the development of the graphical interface of the Navigator we have used the Processing programming language (developed by Ben Fry and Casey Reas). The code created in Processing follows the following block diagram:

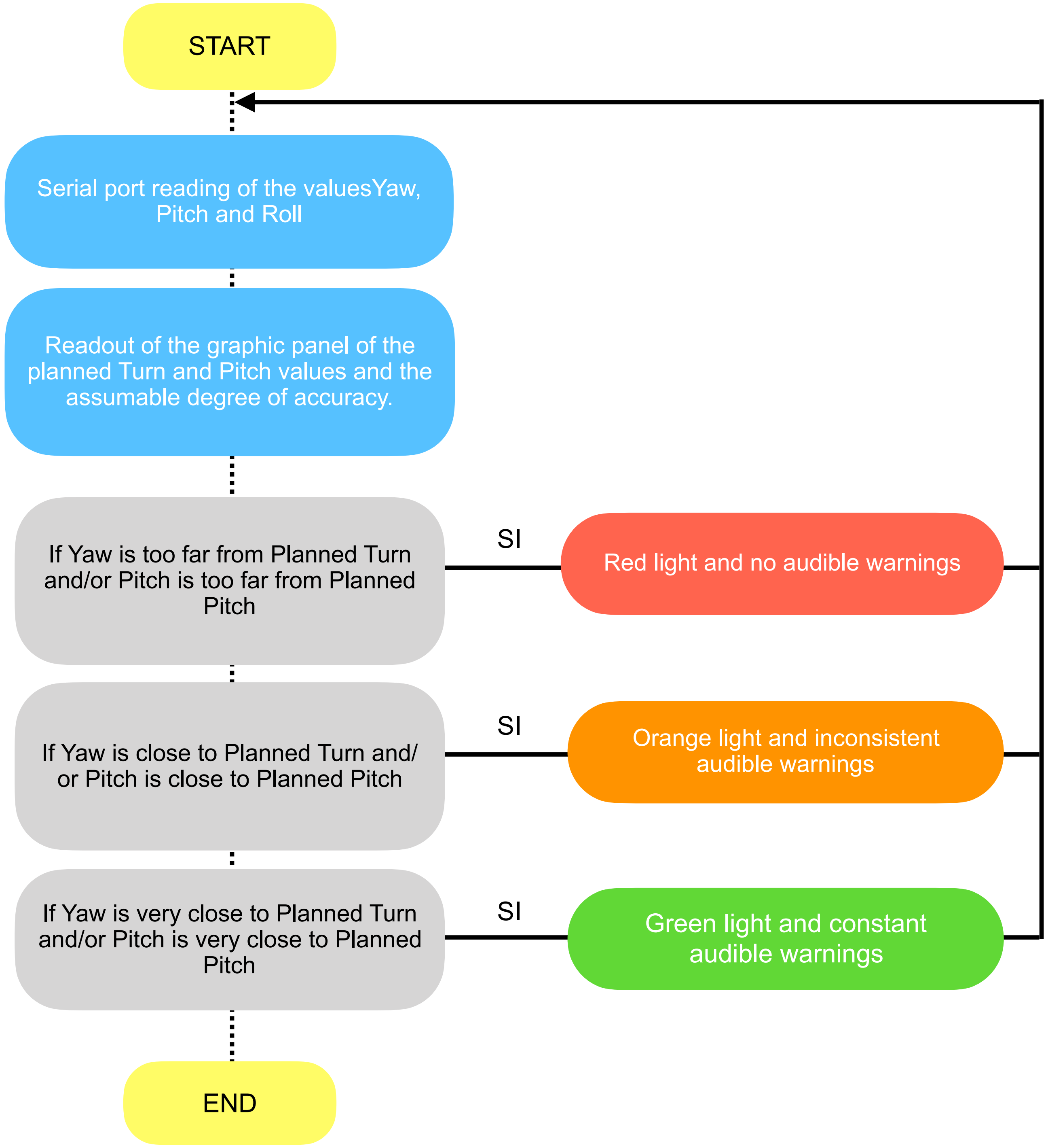

Processing is an integrated, open source, Java®-based (Sun Microsystems, California, USA) programming language and development environment that is easy to use and serves as a medium for teaching and producing multimedia and interactive digital design projects.

As a result, we obtain a graphical interface through which the actual navigation will take place. It has the following visual appearance:

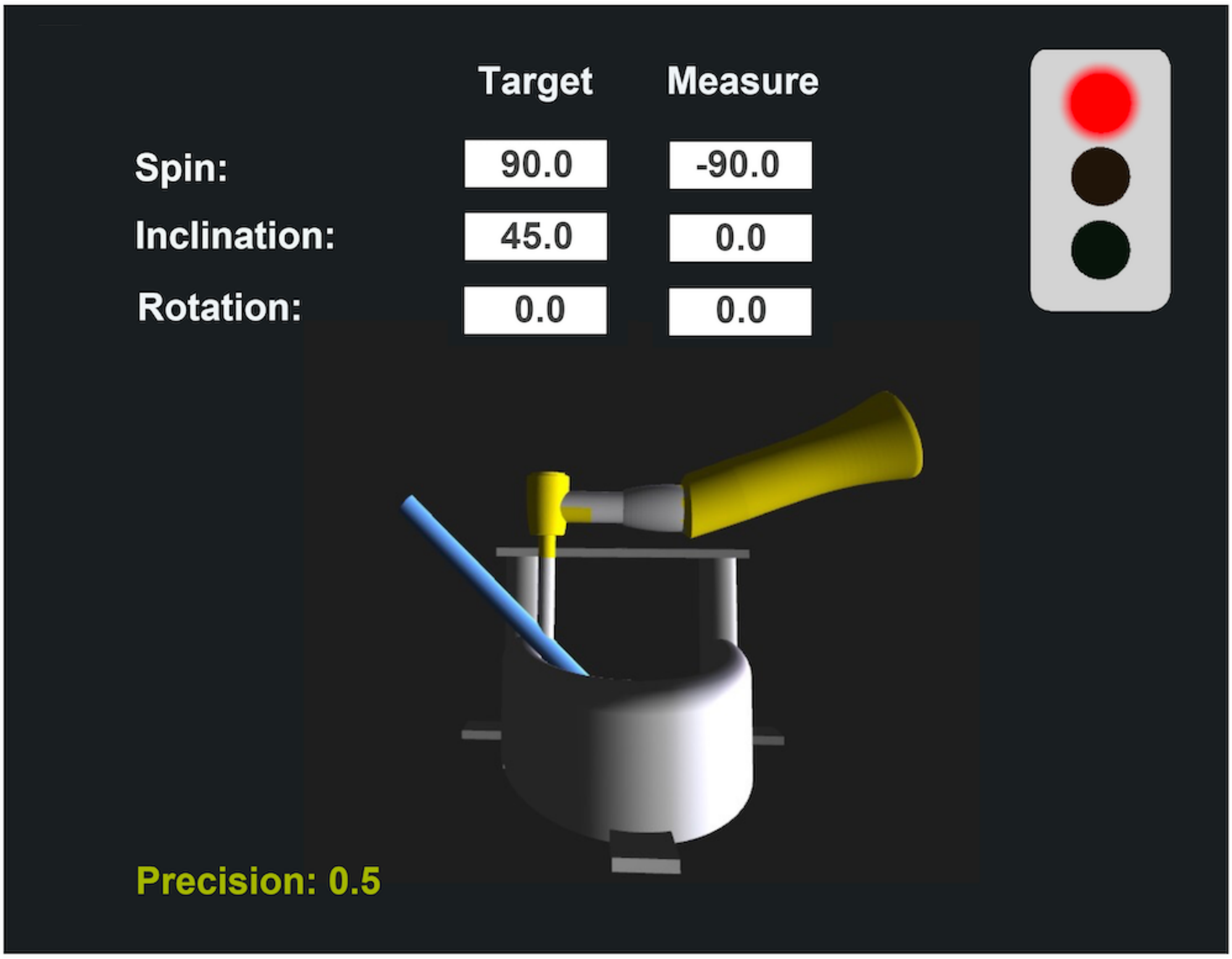

Supplement: S4 File — (PDF) [file pone.0255481.s004.pdf]
